# Estimating the causal influence of body mass index on risk of Parkinson disease: A Mendelian randomisation study

**DOI:** 10.1371/journal.pmed.1002314

**Published:** 2017-06-13

**Authors:** Alastair J. Noyce, Demis A. Kia, Gibran Hemani, Aude Nicolas, T. Ryan Price, Eduardo De Pablo-Fernandez, Philip C. Haycock, Patrick A. Lewis, Thomas Foltynie, George Davey Smith, International Parkinson Disease Genomics Consortium, Anette Schrag, Andrew J. Lees, John Hardy, Andrew Singleton, Mike A. Nalls, Neil Pearce, Debbie A. Lawlor, Nicholas W. Wood

**Affiliations:** 1 Department of Molecular Neuroscience, UCL Institute of Neurology, University College London, London, United Kingdom; 2 Centre for Neuroscience and Trauma, Barts and The London School of Medicine and Dentistry, Queen Mary University of London, London, United Kingdom; 3 MRC Integrative Epidemiology Unit, University of Bristol, Bristol, United Kingdom; 4 School of Social and Community Medicine, University of Bristol, Bristol, United Kingdom; 5 Laboratory for Neurogenetics, National Institute on Aging National Institutes of Health, Bethesda, Maryland, United States of America; 6 School of Pharmacy, University of Reading, Reading, United Kingdom; 7 Sobell Department of Motor Neuroscience and Movement Disorders, UCL Institute of Neurology, University College London, London, United Kingdom; 8 Department of Clinical Neurosciences, UCL Institute of Neurology, University College London, London, United Kingdom; 9 Data Tecnica International, Glen Echo, Maryland, United States of America; 10 Department of Medical Statistics, London School of Hygiene & Tropical Medicine, London, United Kingdom; University of Cambridge, UNITED KINGDOM

## Abstract

**Background:**

Both positive and negative associations between higher body mass index (BMI) and Parkinson disease (PD) have been reported in observational studies, but it has been difficult to establish causality because of the possibility of residual confounding or reverse causation. To our knowledge, Mendelian randomisation (MR)—the use of genetic instrumental variables (IVs) to explore causal effects—has not previously been used to test the effect of BMI on PD.

**Methods and findings:**

Two-sample MR was undertaken using genome-wide association (GWA) study data. The associations between the genetic instruments and BMI were obtained from the GIANT consortium and consisted of the per-allele difference in mean BMI for 77 independent variants that reached genome-wide significance. The per-allele difference in log-odds of PD for each of these variants was estimated from a recent meta-analysis, which included 13,708 cases of PD and 95,282 controls. The inverse-variance weighted method was used to estimate a pooled odds ratio (OR) for the effect of a 5-kg/m^2^ higher BMI on PD. Evidence of directional pleiotropy averaged across all variants was sought using MR–Egger regression. Frailty simulations were used to assess whether causal associations were affected by mortality selection.

A combined genetic IV expected to confer a lifetime exposure of 5-kg/m^2^ higher BMI was associated with a lower risk of PD (OR 0.82, 95% CI 0.69–0.98). MR–Egger regression gave similar results, suggesting that directional pleiotropy was unlikely to be biasing the result (intercept 0.002; *p* = 0.654). However, the apparent protective influence of higher BMI could be at least partially induced by survival bias in the PD GWA study, as demonstrated by frailty simulations. Other important limitations of this application of MR include the inability to analyse non-linear associations, to undertake subgroup analyses, and to gain mechanistic insights.

**Conclusions:**

In this large study using two-sample MR, we found that variants known to influence BMI had effects on PD in a manner consistent with higher BMI leading to lower risk of PD. The mechanism underlying this apparent protective effect warrants further study.

## Abstract 2

Using Mendelian randomization, Nicholas Wood and colleagues examine the association between genetically conferred risk of higher BMI and the risk of Parkinson Disease.

## Author summary

**Why was this study done?:**

- There have been a number of conflicting observational studies describing associations between higher body mass index (BMI) and the future risk of Parkinson disease (PD). The most recent meta-analysis of cohort studies suggested that, overall, there was no effect.
- Observational studies have limitations, such that causal associations may not be reliably ascertained.
- This is the first time, to our knowledge, Mendelian randomisation has been used to study a potential causal association between genetically determined higher BMI and risk of PD.

**What did the researchers do and find?:**

- We used two-sample Mendelian randomisation, in which the relationship between genetic variants and BMI was ascertained from one study, and the relationship between the same genetic variants and PD was ascertained from a second study. Using this method, one can determine the estimated change in risk of PD per unit change in BMI.
- Our analysis indicated a potential causal influence of higher BMI protecting against PD.
- A limitation of the approach is that individuals who have higher BMI have a higher risk of earlier mortality, and therefore individuals with lower BMI may be over-represented amongst individuals diagnosed with PD. This “frailty effect” could at least partially account for the estimated causal effect.

**What do these findings mean?:**

- Further investigation, including exploring potential mechanisms, is warranted.
- Although our results suggest that higher BMI is potentially protective against PD, the negative health impacts of raising BMI are likely to be significant, and should be taken into account.

## Introduction

The risk of many disease outcomes increases as body mass index (BMI) increases, including cardiovascular disease and cancer [1]. Low BMI has also been associated with excess mortality (e.g., due to lung disease), but it is unknown whether these relationships are causal [1,2].

Parkinson disease (PD) is the second most common neurodegenerative disease worldwide [3]. The population burden is increasing for PD, relative to other diseases, even after population ageing is taken into account, and there is no cure [4]. A wide range of potential risk factors for PD have been described, mainly in observational studies [5,6].

The role of BMI in risk of PD is unclear, with observational studies providing conflicting results. A meta-analysis of case–control studies found significantly lower BMI in patients with PD compared with controls, but reverse causality could explain these findings (i.e., weight loss as a result of PD) [7]. A nested case–control study suggested that weight loss may precede the clinical diagnosis of PD [8]. Other studies have found negative associations between BMI and PD, some have found apparent null associations, and one has found a positive association [9–12].

Ten cohort studies were recently meta-analysed, and the pooled odds ratio (OR) for the association of a 5-kg/m^2^ higher BMI with risk of PD was 1.00 (95% 0.89–1.12) [13]. However, there was substantial heterogeneity between studies (*I*^2^ = 65%; *p =* 0.003), and individual studies may have been affected by residual confounding, bias, and, again, reverse causality (the latter can occur even in cohort studies, because PD has a long prodromal phase prior to diagnosis).

**Fig 1:**
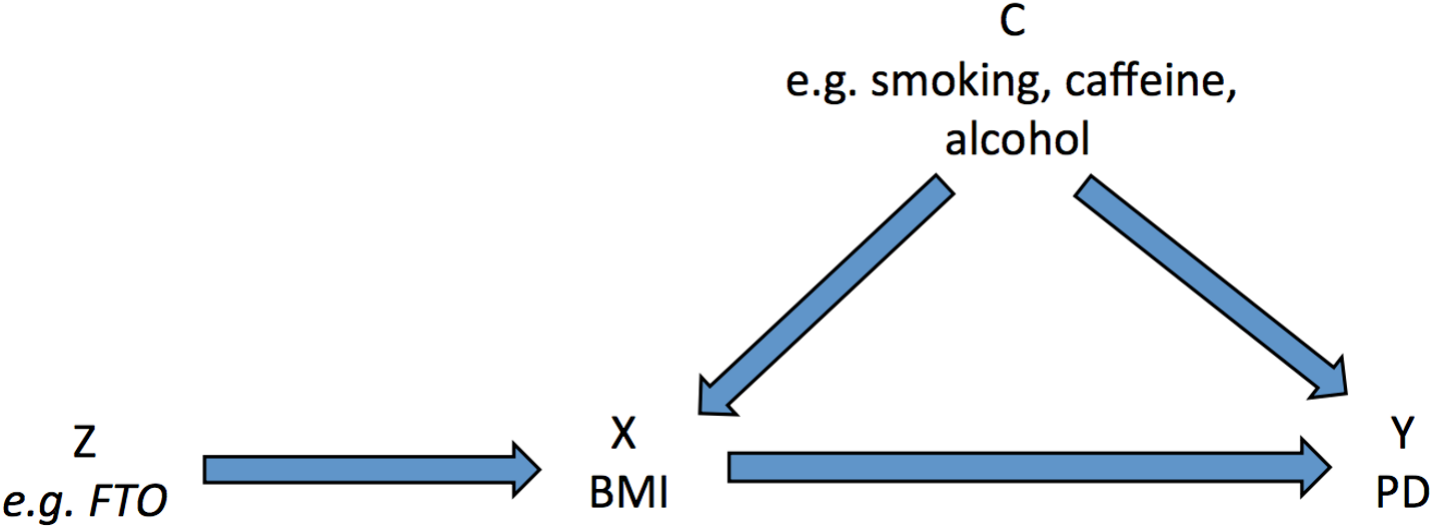
Directed acyclic graph of instrumental variable analysis using genetic variants as proxies for environmental exposures (adapted from Lawlor et al. [14]). Genetic variants (*Z*) associated with an exposure such as BMI (*X*) can be used as proxies to determine the effect of the exposure (*X*) on the outcome (*Y*). The three IV assumptions are indicated by arrows or the absence of arrows: (1) the IV in this schematic (*FTO* gene variant) is robustly associated with the exposure; (2) the IV is not associated with confounding factors (*C*); and (3) there is no alternative way that the IV affects the outcome other than via the exposure. BMI, body mass index; IV, instrumental variable; PD, Parkinson disease.

Mendelian randomisation (MR) is an instrumental variable (IV)–based method to infer causality in observational studies [14]. Gene variants that are associated with intermediate phenotypes or environmental exposures can be used as IVs to estimate the effect of the exposure on a disease outcome (see Fig 1). Random assortment of gene variants during gametogenesis means that potential confounding factors (observed and unobserved) are more likely to be evenly distributed, and the effect estimate that results from IV analysis is likely to be free from residual confounding and reverse causality. Given the difficulties in identifying causal risk factors for PD, MR has great potential to advance aetiological knowledge and identify putative therapeutic targets [15]. Methods are evolving rapidly as more in silico datasets become available in the form of extensive genetic data for a huge range of phenotypes and outcomes. Two-sample MR involves measuring variant–exposure associations in one dataset and variant–outcome associations in a second [16]. However, MR relies on certain assumptions:

1. **The IV is robustly associated with the exposure of interest.** This assumption can be checked by calculating an *F* statistic and *R*^2^ value. The IV may explain only a tiny amount of variance in the exposure (*R*^2^), and therefore studies often have to be large. As a result of genome-wide association (GWA) studies, there are increasing numbers of genetic variants that account for the variance in a range of exposures and outcomes, allowing instruments of greater strength to be constructed.
2. **The IV is independent of known confounders.** In MR studies that have individual-level data, one can check for known confounders and compare the frequency of these between the two levels of the IV. In two-sample MR, the absence of individual-level data about potential confounders can hamper the ability to test this assumption.
3. **The IV is independent of the outcome, given the exposure and confounders.** In other words, there must be no alternative path, other than via the exposure, that the IV influences the outcome. This is known as the exclusion restriction criterion. One situation that violates this assumption is horizontal pleiotropy, in which there are alternative pathways through which the IV affects the outcome.

Here we describe the use of two-sample MR to estimate the causal association between genetically conferred variance in BMI and risk of PD.

## Methods

Two-sample MR was undertaken using GWA study data. Ethical approval was not sought for this specific project because all data came from the summary statistics of published GWA studies, and no individual-level data were used.

### Genetic variant instruments for body mass index

SNPs from the largest GWA study of BMI to date were identified from the 2015 summary statistic files from the GIANT (Genetic Investigation of Anthropometric Traits) consortium (http://portals.broadinstitute.org/collaboration/giant/index.php/GIANT_consortium) [17]. Data on major and minor alleles for each SNP (after imputation, 2,554,637 variants in 339,224 individuals of European descent), along with allele frequencies, beta coefficients for allele dose and 5-kg/m^2^ change in BMI (i.e., the change in BMI on a 5-kg/m^2^ scale per effect allele), *p-*values, and standard errors (SEs) were extracted.

This large number of variants was “clumped” to obtain a set of index SNPs that were independent of each other and associated with BMI at the genome-wide significance level (i.e., *p*-value *<* 5 × 10^−8^). Index SNPs were identified by ranking BMI associations from the smallest to largest *p-*value (but still with a cutoff value of *p =* 5 × 10^−8^). Clumped SNPs were those in linkage disequilibrium (LD) with index SNPs (*R*^2^ threshold of 0.001) or within 10,000 kb physical distance, based on a reference dataset (1000 Genomes Project; http://www.internationalgenome.org/). Hence, each index SNP represented a number of clumped SNPs that were all associated with or near to the index SNP, and the index SNPs were all independent of one another (according to the parameters defined here). Independence of index SNPs is important because bias can be introduced if there is LD between them and can result to over-precise estimates in subsequent analysis. Standard code for clumping is available on the PLINK website (http://zzz.bwh.harvard.edu/plink/clump.shtml), and further information is provided in S1 Appendix.

After clumping, there were 78 independent SNPs that were associated with BMI (*p <* 5 × 10^−8^), and together these explained 2.2% of the variance in BMI (*R*^2^ = 0.022), as calculated from the summary files from the GIANT consortium [17]. To test the statistical significance of the association of the instrument with BMI, an *F* statistic was calculated using the following formula, where *k* is the number of variants and *n* is the sample size:

$$F=\frac{R^{2}\left(n-1-k\right)}{\left(1–R^{2}\right)\times k}$$

### Association of body mass index genetic variants with Parkinson disease

PD genotyping data were from the most recent meta-analysis of GWA studies in PD, which related 7,782,514 genetic variants (after imputation) to PD, in up to 13,708 PD cases and 95,282 controls from 15 independent GWA datasets of individuals of European descent; the meta-analysis was undertaken by the International Parkinson Disease Genomics Consortium (IPDGC; http://pdgenetics.org/) [18]. We extracted the per-allele log-OR of PD together with its SE for each of the independent, genome-wide significant BMI SNPs identified from the GIANT consortium. It was possible to do this for 76 of the 78 BMI SNPs; for one SNP (rs1016287) a suitable proxy in high LD was available (rs887912; *R*^2^ = 1.0), but for another SNP (rs2245368), no suitable proxy (with *R*^2^ > 0.9) could be found, leaving 77 variants available for IV analysis. Three SNPs (rs1558902, rs17001654, and rs4256980) were palindromic, resulting in potential strand ambiguity. Allele frequencies for these were compared between the BMI and PD datasets to ensure that effect estimates were recorded with respect to the same effect allele.

### Two-sample Mendelian randomisation methods

Two-sample MR was undertaken using previously described methods and as summarised below [19,20]. Wald ratios (β_IV_) were calculated for each of the 77 SNPs by dividing the per-allele log-OR of PD (β_ZY_) by the per-allele difference in mean BMI for each SNP (β_ZX_):

$$\beta_{IV}=\beta_{ZY}/\beta_{ZX}$$

95% confidence intervals (95% CIs) were calculated from the SE of each Wald ratio, which was derived from the SE of the variant–outcome association divided by the variant–exposure association. Conventional linear regression analysis of the variant–exposure association and variant–outcome association for each instrument was undertaken and weighted by inverse variance. The regression was constrained to pass through the origin, which forces the assumption that when the exposure has a value of zero, the outcome is also zero. This is known as the inverse-variance weighted (IVW) method, and the point estimate is equal to that derived from fixed-effect meta-analysis. The IVW method assumes that all variants are valid IVs.

Individual Wald ratios and 95% CIs were compiled in a forest plot. Heterogeneity in Wald ratios was tested using Cochran’s *Q* and quantified using the *I*^2^ test. A “leave one out” sensitivity analysis was undertaken to identify variants with disproportionate effects, again using the IVW method.

To establish that violations of the third assumption of IV analysis were not biasing the estimate of the causal association (i.e., that there was not an aggregate unbalanced horizontal pleiotropic effect), MR–Egger regression was used. MR–Egger regression is similar to IVW regression, except that the intercept is not constrained to pass through the origin [20]. Substantial heterogeneity in the IVW estimate indicates that alternative pathways may exist from some of the SNPs to the outcome (known as horizontal pleiotropy), but this does not necessarily bias the estimate. However, a non-zero intercept from the MR–Egger regression suggests that pleiotropic effects tend to be in the direction of the intercept term, which will bias IVW estimates. A statistical hypothesis test can be performed to see if there is evidence of the intercept being different from zero, indicating overall unbalanced pleiotropy. The slope of the MR–Egger regression provides the estimate of the effect of BMI on PD when the third assumption is relaxed. The estimate is correct providing an additional assumption holds, the InSIDE (instrument strength independent of direct effect) assumption. This assumption states that the associations between genetic variants and the exposure are independent of the direct effects of the variants on the outcome [20].

In the absence of unbalanced pleiotropy, the IV estimates for individual SNPs ought to be symmetrically distributed around the point estimate, which, if centred on the IVW estimate, suggests that the result is not systematically biased. This can be demonstrated using a funnel plot of the individual variant effects plotted against the inverse of their SE.

We also repeated the IVW and MR–Egger analyses using the original 97 independent variants reported by the GIANT consortium (i.e., without the clumping step described above) [17]. Similar to the analyses with the 77 clumped variants, two variants were not available in the IPDGC data. The same proxy was used for one, and no proxy was found for the other, leaving 96 variants in the instrument.

Power calculations were undertaken using the proportion of variance in BMI explained by the 77 independent SNPs (*R*^2^ = 0.022) and the methods described by Brion and colleagues [21]. There was 92% power to detect a relative 20% difference in PD risk for a 5-kg/m^2^ difference in BMI (i.e., an OR of at least 0.80 or 1.20) in the IPDGC cases and controls with an alpha of 5% (*p-*value ≤ 0.05). Power reduced to 38% to detect a relative difference in risk of 10%. These power calculations assumed no heterogeneity.

### Frailty analysis

Studies of disorders that are strongly influenced by age (such as neurodegenerative disease) may be prone to bias if selective mortality has occurred [22]. For example, if people with high BMI died prematurely before being diagnosed with PD, then bias could occur because individuals with lower BMI live longer, resulting in a greater risk of being diagnosed with PD. Such an induced association would not reflect any biological link between BMI and PD. We performed simulations to estimate the likely effect that our MR analysis would show due to survival bias, assuming that BMI was not related to PD. The objective was to see if the likely magnitude of the survival bias was large enough to explain the MR results estimated from the real data. We performed simulations where a large sample (*n* = 500,000) was generated with data on BMI, SNPs influencing BMI, age, mortality status, and PD status. The variables were related using the following model:

1. Individuals were randomly assigned genotypes for each of 77 SNPs, with allele frequencies corresponding those in the MR analysis.
2. BMI values for each individual were simulated using their genotype values and the effect size (in standard deviation units) of each SNP, and a random value to create a population variance of one.
3. An age variable was generated for all individuals to match the distribution of ages in the PD meta-analysis.
4. Alive/dead status was simulated for individuals as a function of their age and BMI. Baseline age-related mortality rates and mortality rates for varying BMI levels were obtained from Davey Smith and colleagues [2]. These were used to generate a Gompertz–Makeham mortality curve, and individuals had their alive/dead status sampled as a function of the probability of death due to the survival curve.
5. PD status was simulated as a function of age-related diagnosis rates obtained from Driver et al. [23]; therefore, PD status was a function of age only, and unrelated to BMI level.
6. A subset of 13,708 simulated individuals with PD and 95,282 without PD, all of whom survived the mortality function, were then retained, based on the distribution of ages of individuals in the Nalls et al. PD GWA study [18]. Observational associations and MR were performed on these individuals to gauge the extent to which an association between BMI and PD was induced artificially by frailty effects.
7. The entire process was repeated 1,000 times to obtain a distribution of the effect size that was due to frailty effects only.

Full details about the simulations are available in S1 Appendix.

All analyses were undertaken in R (version 3.2.3).

## Results

Table 1 shows the 77 variants selected for the analysis, with the effect alleles and frequencies, the magnitude of the effect on BMI, and strength of the association with PD. Further information on each variant, including regional genes and functions, are given in S1 and S2 Tables in S1 Appendix. The per-allele results of the IV analysis are provided in S3 Table in S1 Appendix. The *F* statistic for the instrument and its association with BMI was 99, which is large. This means that weak instrument bias was unlikely.

**Table 1:** Variants and effect alleles with frequencies and magnitude of effect on BMI and strength of association with PD.

SNP	Chr	BP	EA	Non-EA	EA freq*	EA BMI beta	SE BMI beta	*p*-Value BMI association	EA PD log-OR	SE PD log-OR	*p*-Value PD association
rs17001654	4	77348592	G	C	0.154	0.030	0.005	7.76 × 10^−09^	−0.077	0.024	0.0014
rs13107325	4	103407732	T	C	0.072	0.047	0.007	1.83 × 10^−12^	−0.109	0.032	0.0007
rs4787491	16	29922838	G	A	0.509	0.015	0.003	2.70 × 10^−08^	−0.031	0.016	0.0508
rs4740619	9	15624326	T	C	0.540	0.017	0.003	4.56 × 10^−09^	−0.029	0.017	0.0777
rs2820292	1	200050910	C	A	0.547	0.018	0.003	1.83 × 10^−10^	−0.031	0.016	0.0587
rs1808579	18	19358886	C	T	0.534	0.016	0.003	4.17 × 10^−08^	−0.027	0.016	0.0913
rs3888190	16	28796987	A	C	0.397	0.031	0.003	3.14 × 10^−23^	−0.045	0.017	0.0076
rs2033732	8	85242264	C	T	0.749	0.018	0.003	4.89 × 10^−08^	−0.023	0.019	0.2352
rs6465468	7	95007450	T	G	0.300	0.016	0.003	4.98 × 10^−08^	−0.020	0.019	0.2844
rs12401738	1	78219349	A	G	0.347	0.020	0.003	1.15 × 10^−10^	−0.023	0.017	0.1812
rs1000940	17	5223976	G	A	0.320	0.018	0.003	1.28 × 10^−08^	−0.02	0.017	0.2477
rs6091540	20	50521269	C	T	0.725	0.019	0.003	2.15 × 10^−11^	−0.02	0.018	0.2659
rs11727676	4	145878514	T	C	0.911	0.037	0.006	2.55 × 10^−08^	−0.037	0.042	0.3745
rs16851483	3	142758126	T	G	0.066	0.048	0.008	3.55 × 10^−10^	−0.046	0.033	0.1647
rs10968576	9	28404339	G	A	0.315	0.025	0.003	6.61 × 10^−14^	−0.024	0.017	0.1669
rs10132280	14	24998019	C	A	0.674	0.022	0.003	1.14 × 10^−11^	−0.021	0.018	0.2513
rs492400	2	219057996	C	T	0.426	0.015	0.003	6.78 × 10^−09^	−0.014	0.017	0.4062
rs7899106	10	87400884	G	A	0.057	0.038	0.007	2.96 × 10^−08^	−0.034	0.039	0.3811
rs1441264	13	78478920	A	G	0.613	0.017	0.003	2.96 × 10^−08^	−0.015	0.016	0.3631
rs3101336	1	72523773	C	T	0.611	0.032	0.003	2.66 × 10^−26^	−0.026	0.016	0.1203
rs13078960	3	85890280	G	T	0.193	0.029	0.004	1.74 × 10^−14^	−0.022	0.020	0.2624
rs17094222	10	102385430	C	T	0.209	0.025	0.004	5.94 × 10^−11^	−0.019	0.020	0.3432
rs11165643	1	96696685	T	C	0.574	0.022	0.003	2.07 × 10^−12^	−0.016	0.016	0.3277
rs12940622	17	76230166	G	A	0.572	0.018	0.003	2.49 × 10^−09^	−0.012	0.016	0.4607
rs11030104	11	27641093	A	G	0.791	0.042	0.004	5.56 × 10^−28^	−0.027	0.020	0.1780
rs7599312	2	213121476	G	A	0.721	0.021	0.003	1.17 × 10^−10^	−0.013	0.018	0.4813
rs6567160	18	55980115	C	T	0.236	0.056	0.004	3.93 × 10^−53^	−0.03	0.019	0.1144
rs2176040	2	226801046	A	G	0.362	0.015	0.003	9.99 × 10^−09^	−0.008	0.017	0.6443
rs1516725	3	187306698	C	T	0.869	0.045	0.004	1.89 × 10^−22^	−0.022	0.024	0.3480
rs17724992	19	18315825	A	G	0.743	0.020	0.003	3.42 × 10^−08^	−0.009	0.018	0.6166
rs7715256	5	153518086	G	T	0.422	0.017	0.003	8.85 × 10^−09^	−0.008	0.016	0.6363
rs12429545	13	53000207	A	G	0.135	0.032	0.004	1.09 × 10^−12^	−0.011	0.024	0.6440
rs1558902	16	52361075	A	T	0.409	0.081	0.003	7.51 × 10^−153^	−0.022	0.017	0.1859
rs17203016	2	207963763	G	A	0.195	0.021	0.004	3.41 × 10^−08^	−0.006	0.021	0.7884
rs657452	1	49362434	A	G	0.397	0.023	0.003	5.48 × 10^−13^	−0.006	0.017	0.7212
rs1460676	2	164275935	C	T	0.179	0.021	0.004	4.98 × 10^−08^	−0.005	0.022	0.8204
rs6804842	3	25081441	G	A	0.569	0.018	0.003	2.48 × 10^−09^	−0.004	0.016	0.7945
rs4256980	11	8630515	G	C	0.638	0.021	0.003	2.90 × 10^−11^	−0.005	0.017	0.7876
rs1528435	15	65864222	T	C	0.631	0.018	0.003	1.20 × 10^−08^	−0.004	0.017	0.8292
rs16951275	2	181259207	T	C	0.771	0.030	0.004	1.91 × 10^−17^	−0.006	0.019	0.7585
rs10182181	2	25003800	G	A	0.468	0.031	0.003	8.78 × 10^−24^	−0.006	0.016	0.7275
rs17024393	1	109956211	C	T	0.043	0.061	0.008	7.03 × 10^−14^	−0.01	0.049	0.8327
rs3817334	11	47607569	T	C	0.401	0.026	0.003	5.15 × 10^−17^	−0.004	0.016	0.8003
rs1928295	9	119418304	T	C	0.550	0.018	0.003	7.91 × 10^−10^	−0.003	0.016	0.8747
rs2112347	5	75050998	T	G	0.621	0.025	0.003	6.19 × 10^−17^	−0.003	0.022	0.9055
rs2365389	3	61211502	C	T	0.572	0.020	0.003	1.63 × 10^−10^	−0.001	0.017	0.9654
rs758747	16	3567359	T	C	0.280	0.023	0.004	7.47 × 10^−10^	0.000	0.018	0.9996
rs13201877	6	137717234	G	A	0.140	0.024	0.004	4.29 × 10^−08^	0.003	0.026	0.9138
rs7138803	12	48533735	A	G	0.379	0.032	0.003	8.15 × 10^−24^	0.005	0.017	0.7605
rs2287019	19	50894012	C	T	0.806	0.035	0.004	4.59 × 10^−18^	0.007	0.020	0.7198
rs2207139	6	50953449	G	A	0.176	0.045	0.004	4.13 × 10^−29^	0.011	0.021	0.6064
rs17405819	8	76969139	T	C	0.702	0.022	0.003	2.07 × 10^−11^	0.006	0.017	0.7158
rs10938397	4	44877284	G	A	0.428	0.040	0.003	3.21 × 10^−38^	0.014	0.016	0.4092
rs29941	14	78969207	G	A	0.670	0.018	0.003	2.41 × 10^−08^	0.006	0.017	0.7122
rs7141420	19	39001372	T	C	0.529	0.023	0.003	1.23 × 10^−14^	0.008	0.017	0.6259
rs2033529	6	40456631	G	A	0.289	0.018	0.003	1.39 × 10^−08^	0.006	0.018	0.7176
rs6477694	9	110972163	C	T	0.371	0.017	0.003	2.67 × 10^−08^	0.006	0.017	0.7016
rs13021737	2	622348	G	A	0.830	0.060	0.004	1.11 × 10^−50^	0.023	0.021	0.2741
rs1167827	7	75001105	G	A	0.557	0.020	0.003	6.33 × 10^−10^	0.009	0.016	0.6014
rs7903146	10	114748339	C	T	0.713	0.024	0.003	1.11 × 10^−11^	0.011	0.018	0.5310
rs543874	1	176156103	G	A	0.195	0.050	0.004	2.62 × 10^−35^	0.025	0.020	0.2265
rs205262	6	34671142	G	A	0.285	0.021	0.003	1.75 × 10^−10^	0.011	0.018	0.5442
rs11057405	12	121347850	G	A	0.902	0.030	0.005	2.02 × 10^−08^	0.018	0.030	0.5438
rs7239883	18	38401669	G	A	0.394	0.015	0.003	1.51 × 10^−08^	0.01	0.016	0.5618
rs2836754	21	39213610	C	T	0.599	0.017	0.003	1.61 × 10^−08^	0.011	0.017	0.5139
rs12286929	11	114527614	G	A	0.523	0.021	0.003	1.31 × 10^−12^	0.014	0.016	0.3858
rs2176598	11	43820854	T	C	0.256	0.019	0.003	2.97 × 10^−08^	0.013	0.018	0.4701
rs3736485	15	49535902	A	G	0.461	0.016	0.003	7.41 × 10^−09^	0.013	0.016	0.4350
rs10733682	9	128500735	A	G	0.475	0.019	0.003	1.83 × 10^−08^	0.017	0.016	0.2755
rs13191362	6	162953340	A	G	0.880	0.029	0.005	7.34 × 10^−09^	0.035	0.025	0.1626
rs9374842	6	120227364	T	C	0.748	0.020	0.003	2.67 × 10^−08^	0.026	0.019	0.1726
rs887912	2	59075742	T	C	0.317	0.023	0.003	2.75 × 10^−11^	0.031	0.018	0.0738
rs9540493	13	65103705	A	G	0.464	0.018	0.003	4.97 × 10^−08^	0.025	0.017	0.1389
rs977747	1	47457264	C	G	0.403	0.017	0.003	2.18 × 10^−08^	0.024	0.016	0.1368
rs2121279	2	142759755	T	C	0.150	0.024	0.004	2.31 × 10^−08^	0.037	0.024	0.1217
rs9400239	6	109084356	C	T	0.673	0.017	0.003	1.61 × 10^−08^	0.028	0.018	0.1180
rs3849570	3	81874802	A	C	0.362	0.018	0.003	2.60 × 10^−08^	0.030	0.017	0.0815

*Frequency of effect allele in Locke et al. [17].

BMI, body mass index; BP, base pair position; Chr, chromosome; EA, effect allele; OR, odds ratio; PD, Parkinson disease; SE, standard error.

**Fig 2:**
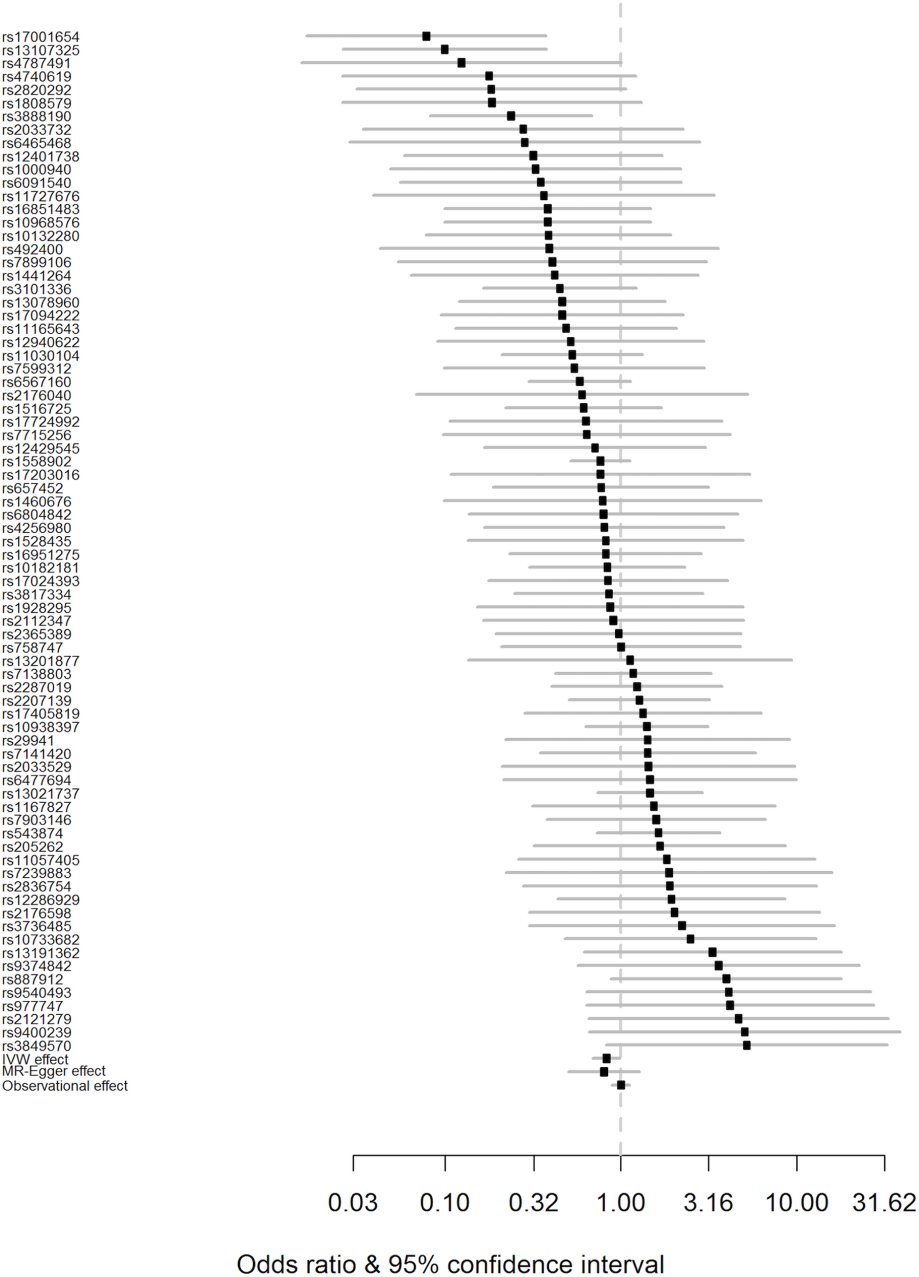
Forest plot of Wald ratios and 95% CIs generated from clumped SNPs associated with BMI. Odds ratios for individual SNPs are listed according to magnitude of effect in the instrumental variable analysis and are presented with pooled effects using the IVW method and MR–Egger regression. The most recent meta-analysis of observational studies is also plotted (Wang et al. [13]). Squares represent the point estimate, and the bars are the 95% confidence intervals. There was weak evidence of heterogeneity (*Q* statistic = 95.5; *I*^2^ = 20.4%; *p* = 0.065). IVW, inverse-variance weighted; MR, Mendelian randomisation.

Using the IVW method to pool results from individual SNPs, genetically conferred higher BMI was found to be associated with a reduced risk of PD. The OR of PD per 5-kg/m^2^ higher BMI was 0.82 (95% CI 0.69–0.98; *p =* 0.029). There was minimal evidence of heterogeneity between variants (*Q* statistic = 95.5; *I*^2^ = 20.4%; *p* = 0.065; see Fig 2 and S3 Table in S1 Appendix). For the observed OR of 0.82, the estimated statistical power was 85% when the alpha was set at 5%.

Results from a “leave one out” analysis demonstrated that no single SNP was driving the IVW point estimate (see S4 Table in S1 Appendix). For example, after removal of the rs1558902 SNP, in the *FTO* locus, which has a strong magnitude of association with BMI, the OR of PD per 5-kg/m^2^ higher BMI was 0.84 (95% CI 0.70–1.00).

The effect estimated from MR–Egger regression was 0.76 (95% CI 0.51–1.14; *p =* 0.177), with an intercept of 0.002 (95% CI −0.008 to 0.013; *p =* 0.654). A funnel plot (see S1 Fig in S1 Appendix) suggested that individual variants were symmetrically distributed around the point estimate. Together these findings provide evidence against the possibility that systematic bias in the IVW estimate may have arisen through overall unbalanced horizontal pleiotropy.

We repeated the analysis using the original set of “un-clumped” variants reported by the GIANT consortium (96 variants in total) [17]. This reanalysis did not alter the observed effect of genetically estimated BMI on risk of PD (IVW OR 0.80, 95% CI 0.67–0.97; see S2 Fig in S1 Appendix).

**Fig 3:**
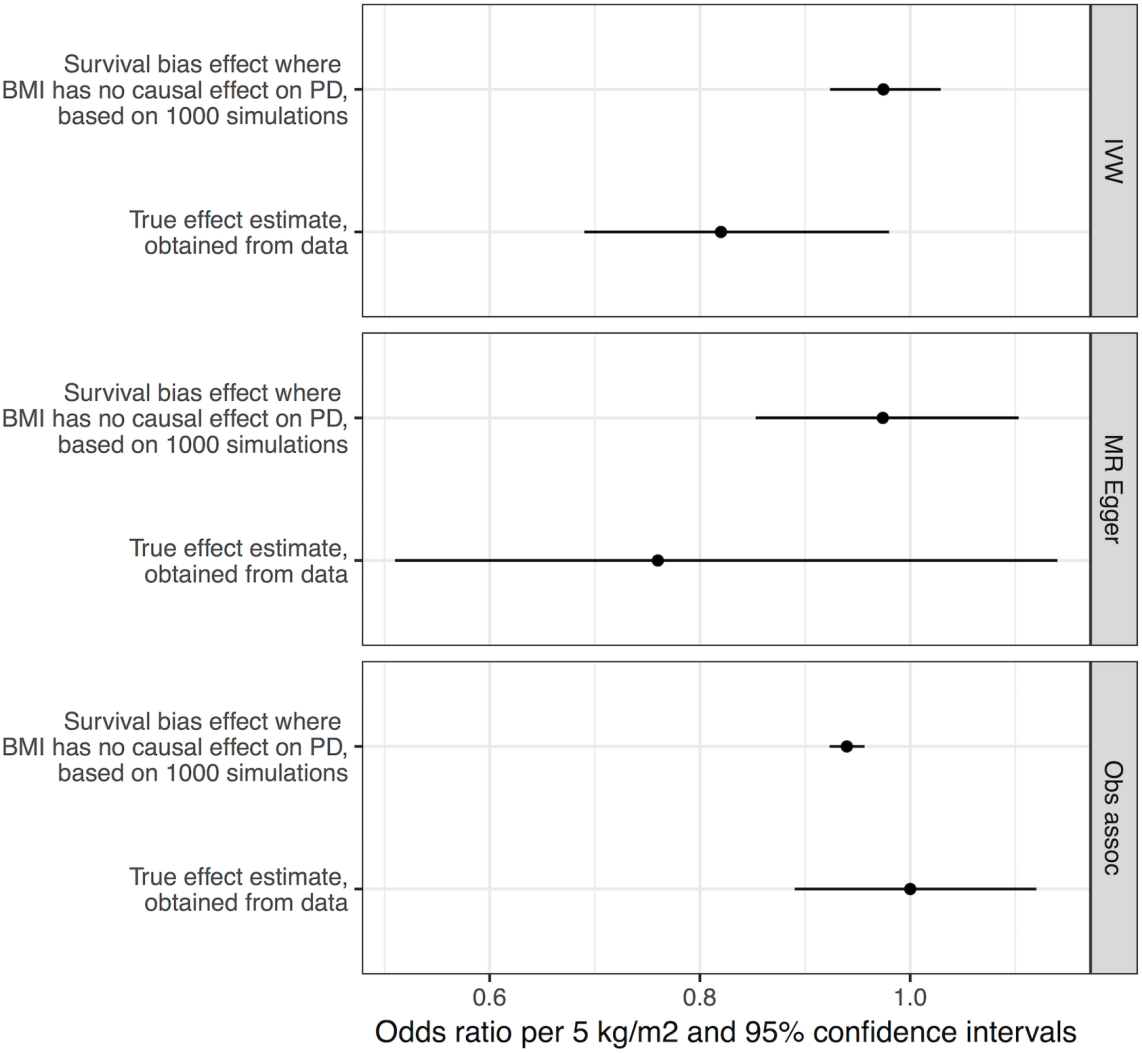
Frailty analysis. We obtained effect estimates from two sources: (1) analysis of the causal influence of BMI on PD using real data and (2) simulations where BMI had no effect on PD, and any apparent effect was due to survival bias alone. This figure shows the comparison of the estimates from these two sources using three different approaches—MR IVW analysis, MR–Egger regression, and observational associations. For the true effect estimates, the horizontal lines denote the 95% confidence intervals; for the results from simulations, the horizontal lines denote 95% confidence intervals obtained from 1,000 simulations. BMI, body mass index; IVW, inverse-variance weighted; MR, Mendelian randomisation; PD, Parkinson disease.

We performed simulations to obtain estimates of effect sizes that would be induced by survival bias alone, under the null hypothesis that BMI is not biologically related to PD. If the effect sizes obtained from these simulations were similar in magnitude to the effect sizes we estimated from the real data, it would suggest that survival bias was sufficient to explain the results. Simulations using empirical BMI- and age-related mortality rates demonstrated that a likely influence of frailty on biasing the MR analysis of BMI and risk of PD was present, but smaller than the estimated inverse influence of BMI on PD from the empirical MR analysis (see Fig 3). The mean induced effect due to frailty alone in the IVW analysis was an OR of 0.97 per 5-kg/m^2^ higher BMI (95% CI 0.92–1.03), compared to our empirical estimate of the OR of PD per 5-kg/m^2^ higher BMI of 0.82 (95% CI 0.69–0.98).

## Discussion

This study provides MR evidence that higher BMI could protect against the risk of developing PD. Simulation studies performed to ascertain the effect of survivor bias showed that survivor bias could be a contributing factor but did not explain all of the effect. To our knowledge, this is the first MR study of this association and was undertaken in a very large sample. Genetic elements expected to lead to a lifetime elevation in BMI of 5 kg/m^2^ were associated with a lower risk of PD, indicating a causal influence of BMI on PD (OR 0.82). Whilst there was minimal evidence of heterogeneity across individual SNP estimates, the MR–Egger analysis supported our main study findings, with an intercept value close to zero and a point estimate for the effect that was similar to that of the main analysis (see S3 Fig in S1 Appendix). MR–Egger regression has less statistical power than an equivalent IVW meta-analysis, and hence the confidence intervals for this analysis were wider and included the null value.

These findings shed additional light on an issue for which the evidence to date has been mixed. Prior to PD diagnosis, observational studies have found conflicting results with regard to the association between BMI and risk of PD [8–12]. The conflicting results may be due to study-specific biases, differential adjustment for confounding factors, and use of different cutoffs to define the exposure (see Table 2). A recent meta-analysis of these and additional studies concluded that there was no robust observational association [13]. There was substantial heterogeneity between the effect estimates of individual studies (*I*^2^ = 65%; *p =* 0.003), raising the question as to whether it was appropriate to perform meta-analysis. Three of these studies did not set out specifically to investigate the effect of BMI on the risk of PD [8,24,25]. Two of the studies showed a clear negative association between higher BMI and the risk of PD [8,11]. Many of the studies used self-report questionnaires to ascertain PD, which is likely to have resulted in under-ascertainment of the outcome and bias towards the null. These factors could have had a strong influence on the overall results of the meta-analysis.

**Table 2:** Examples of previous observational studies of body mass index and Parkinson disease.

Authors and year [reference]	Study design and location	Observed association	Handling of potential sources of bias	Handling of confounding
Ma et al. 2006 [8]	Nested case–control study in Linxian County, China. Risk of reverse causality reduced because measurement of BMI preceded ascertainment of the outcome.	BMI inversely associated with risk of PD, with dose-dependent effect for increasing BMI.	Two-step ascertainment of PD diagnosis and standard clinical criteria to reduce bias. Only definite cases included. Generalisability may be limited by cohort and location.	Cases matched to controls 1:4 by age and sex. Multiple logistic regression analysis modelling BMI, gastric ulcers, meat consumption, and pack-years smoking (all associated with PD in univariate analysis).
Chen et al. 2004 [9]	Health Professionals Follow-up Study and Nurses’ Health Study cohorts, US; excluded cases diagnosed within 3–4 y of baseline.	Baseline BMI not associated with risk of PD, but most recently recorded BMI was inversely associated with PD.	Questionnaire during follow-up included item about diagnosis of PD, with positive responses confirmed by neurological examination. This approach misses undiagnosed cases and biases estimates towards the null value. Generalisability may be limited by cohort composition.	Associations adjusted for age, smoking, caffeine intake, and alcohol intake. Men and women presented separately and in pooled analysis.
Logroscino et al. 2007 [11]	Harvard Alumni Health Study cohort, US; 63,557 person-years of follow-up; excluded cases diagnosed within 4 y of baseline.	Trend for inverse association between BMI measured in 1988 and PD. No association between BMI measured in college and PD.	Self-reported diagnosis of PD (previous validation study found 70% to be correct). Non-differential misclassification of the outcome would bias estimates towards null.	Associations adjusted for age, smoking, caffeine intake, physical activity, and previous cardiovascular disease or cancer.
Hu et al. 2006 [12]	Six independent population surveys, Finland. Mean follow-up 18.8 y; excluded cases diagnosed within 5 y of baseline.	BMI positively associated with PD, with dose-dependent effect for increasing BMI.	PD diagnosis from national drug reimbursement register. May under-ascertain cases with low BMI—differential misclassification—if obese individuals are more likely to contact health system.	Associations adjusted for age, study year, blood pressure, cholesterol level, education, physical activity, smoking, caffeine intake, and alcohol intake. Men and women presented separately and pooled.

BMI, body mass index; PD, Parkinson disease.

Recently, a similar direction and magnitude of effect of higher BMI on risk of PD was reported from the United Kingdom Clinical Practice Research Datalink. In a cohort of nearly 2 million, underweight people had a 15% excess risk of PD compared with normal weight people, and overweight and obese people had a 12% and 17% reduction in risk, respectively [10].

The mechanism through which higher BMI may reduce the risk of PD is not immediately apparent, but researchers have recently reported on possible neuroprotective benefits arising in those with higher BMI, particularly relating to preservation of cognitive function and neural networks, explored using functional imaging [26]. Higher BMI affects levels of circulating and central insulin, which in turn may play a beneficial role with respect to neurodegeneration [27]. Theories such as these require substantial further exploration.

Weight loss is widely recognised in patients with a diagnosis of PD [7]. The mechanisms underlying body weight reduction in this context are easier to understand and are multifactorial, including intrinsic factors of the disease as well as disruption of both peripheral and central regulatory mechanisms of body weight, feeding behaviour, and energy metabolism [28]. Vitally, however, the mechanisms that drive risk of PD may not be the same as those that underlie progression once disease has occurred, and the MR study presented here is designed to investigate only the former.

There are a number of candidate genes (see S1 and S2 Tables in S1 Appendix) that are located in close proximity to SNPs included as IVs, which is intriguing and raises the possibility of pleiotropic pathways (i.e., not via BMI). *BDNF*, coding for Brain-Derived Neurotrophic Factor, is a gene that has previously been implicated in PD, and BDNF has been discussed as a potential therapeutic agent in neurodegenerative disease but has previously been unsuccessful in clinical trials in patients with amyotrophic lateral sclerosis [29–31]. *PARK2*, coding for the Ubiquitin E3 ligase Parkin, is mutated in rare autosomal recessive juvenile-onset parkinsonism cases, and is notable for not having been identified in a GWA study for PD [18,32]. Finally, the *RPTOR* gene (encoding Raptor, a regulator of the kinase mTOR) is of interest given the links between the mTOR pathway, macroautophagy, and PD [33]. For each of these examples, proof that these genes are directly linked to causation at these loci requires further research.

The finding of higher BMI reducing the risk of PD seemingly conflicts with the observation from a cohort study that physical activity may be a protective factor for PD [34]. Again, cohort studies such as this may be biased by confounding or reverse causality, and the association with physical activity may be driven simply by the fact that people with undiagnosed PD may undertake less activity as a result of occult disease. However, evidence of an effect of physical activity on BMI may be weaker than one might intuitively expect [35,36]. There may be true inverse causal associations for both BMI and physical activity with risk of PD, and physical activity may protect primarily through mechanisms that are independent of BMI. An MR study (or randomised controlled trial) of physical activity and risk of PD could be used to explore this further, if robust genetic variants could be identified to account for variability in this behaviour.

An MR study to determine an association between genetically conferred variance in BMI (alongside other potentially modifiable risk factors) and Alzheimer disease was recently reported [37]. The authors observed no similar protective effect to that which we observed for PD, but their instrument used only 32 variants. They did observe a reduction in risk of Alzheimer disease for increasing blood pressure, but did not undertake a similar frailty analysis to that which we conducted.

Our frailty simulations sought to explore the extent to which our MR results could be explained by frailty effects alone. The influence of frailty effects should be an important consideration in epidemiological studies dealing with later life events [22]. We found that the effect of BMI on mortality and the age disparity between the cases and controls in the PD GWA study was unlikely to be sufficient to explain our MR estimate, particularly given that PD has a relatively early onset compared to some other neurological disorders (median age 60 y old) [3].

### Strengths and weaknesses

MR uses an IV approach to assess causal relationships between environmental exposures/intermediate phenotypes and disease outcomes, while minimising or eliminating the possibility of bias due to residual confounding or reverse causality. One key assumption is that the instrument affects PD risk through its effect on a specific phenotype/exposure (here this was BMI), and does not have a direct effect on PD risk independently of this (the exclusion restriction criterion, or assumption number three from the Introduction). We tested this assumption using MR–Egger regression and found no evidence of violation of this assumption.

This analysis was undertaken using summary statistics from the two largest datasets for BMI and PD. The use of different datasets to ascertain variant–exposure and variant–outcome associations, so-called two-sample MR, has methodological advantages over analysis undertaken in a single dataset, but also some limitations [38]. First, the data are available without having to undertake measurement of each association in turn, saving both time and cost. Second, statistical power is high because very large sample sizes can be achieved using this method. Third, if bias did arise in the analysis due to weak instruments, it would tend to be biased towards the null (i.e., give rise to conservative estimates), whereas in one-sample MR the estimate tends to be biased towards the confounded observational study estimate [16]. The first assumption of MR is that the instrument under study is strongly associated with the exposure. Here, the first-stage *F* statistic was large (99), and so weak instrument bias is unlikely. Furthermore, samples are assumed to be completely independent. The cohorts contributing to both the GIANT and IPDGC consortia are listed in S5 and S6 Tables in S1 Appendix. If there was some overlap in the controls from the two consortia, then an *F* statistic of this magnitude would mitigate potential bias.

Our frailty analysis used demographic information to model the induced bias due to the combination of BMI influencing mortality and cases being on average younger than controls in the PD GWA study. Alternative mechanisms of frailty may operate. For example, because BMI reduces longevity, individuals with high BMI who survive longer potentially over-represent unmeasured factors that increase longevity. If these factors, in turn, also influence risk of PD, then bias could be introduced into our MR estimates. This mechanism can influence observational associations also.

One weakness of our approach is that two-sample MR using aggregate data (as done here) does not currently allow one to examine non-linear relationships between exposures and outcomes. The association between BMI and most health-related outcomes tends to be U- or J-shaped, with very low weight and above average weight both resulting in adverse outcomes [1]. Here, in the MR analysis, BMI was modelled as a continuous variable, and linearity assumed. The use of aggregate data also means that we were not able to test differences of effect in subgroups. There is evidence to suggest that the causal effect of BMI on some outcomes, including socioeconomic status, may differ between men and women [39]. Using this design, we cannot test whether effects of BMI on PD differ by sex. In addition, lack of information about potential confounding factors for the relationship of BMI and PD means that associations between the variants and smoking/alcohol behaviours could not be tested (assumption number two from the Introduction). Another limitation of this study is that it does not offer insight into the mechanisms by which BMI is causally related to PD. Finally, two-sample MR assumes that both samples come from the same population, but with no overlap. If they come from separate populations, the magnitude of the estimated causal effect could be biased. We took steps to ensure that both samples were of individuals of European ancestry.

In this large two-sample MR study exploring the association between genetically determined higher BMI and risk of PD, we observed a possible protective effect exerted by a potentially modifiable factor. MR should be seen as one tool to explore causal questions, but not as a definitive answer. However, robust empirical evidence to support a protective role of higher BMI in risk of PD may be hard to obtain. It is plausible that our finding could be replicated in large cohort studies such as the UK Biobank, which gathered information on a wide range of exposures and disease outcomes, along with genotyping data, on more than half a million older people in the UK [40]. Our study offers the best evidence to date that higher BMI may convey relative protection against PD, in support of clinical observation and some of the pre-existing observational studies. However, the apparent protective influence of higher BMI could be at least partially induced by survival bias in the PD GWA study, as demonstrated in the frailty simulations.

Although BMI is a potentially modifiable risk factor for PD, the negative health impacts of raising BMI are likely to be significant, and should be taken into account. Expected negative effects would include increased risk of type 2 diabetes mellitus, ischaemic heart disease, and cancer. Causal associations have been demonstrated between higher BMI and many cancers, and have been explored for BMI and cardiovascular disease [41,42]. Assuming replication of a negative association between BMI and PD, a great deal of further work would be required (including mechanistic insights) before recommending this as a potential intervention against PD, given the wider public health effects.

## Supporting information

S1 Appendix Supplementary materials. (DOCX)

## Abbreviations

BMI: body mass index
GWA: genome-wide association
IPDGC: International Parkinson Disease Genomics Consortium
IV: instrumental variable
IVW: inverse-variance weighted
LD: linkage disequilibrium
MR: Mendelian randomisation
OR: odds ratio
PD: Parkinson disease
SE: standard error
