# Supplementary material for: Estimating the causal influence of body mass index on risk of Parkinson disease: A Mendelian randomisation study
Source: PLoS Med. 2017 Jun 13;14(6):e1002314. doi: 10.1371/journal.pmed.1002314 (PMC5469450; doi:10.1371/journal.pmed.1002314)
Supplement: S1 Appendix — (DOCX) [file pmed.1002314.s001.docx]

**S1 Appendix:**

**Estimating the causal influence of BMI on risk of Parkinson’s disease: a Mendelian randomization study**

**Noyce AJ et al**

**IPDGC consortium members and affiliations:**

Alastair J Noyce (Department of Molecular Neuroscience, UCL, London, UK), Alexis Brice (INSERM, UMR_S975, Université Pierre et Marie Curie-Paris, CNRS, UMR 7225, AP-HP, Pitié-Salpêtrière Hospital, Paris, France), Anamika Giri (Department for Neurodegenerative Diseases, Hertie Institute for Clinical Brain Research, University of Tübingen, and DZNE, German Center for Neurodegenerative Diseases, Tübingen, Germany), Angelika Oehmig (Department for Neurodegenerative Diseases, Hertie Institute for Clinical Brain Research, University of Tübingen, and DZNE, German Center for Neurodegenerative Diseases, Tübingen, Germany), Arianna Tucci (Department of Molecular Neuroscience, UCL Institute of Neurology, London, UK), Aude Nicolas (Laboratory of Neurogenetics, National Institute on Aging, Bethesda, MD, USA), Claudia Schulte (Department for Neurodegenerative Diseases, Hertie Institute for Clinical Brain Research), Mark R Cookson (Laboratory of Neurogenetics, National Institute on Aging, Bethesda, USA), Cornelis Blauwendraat (Laboratory of Neurogenetics, National Institute on Aging, Bethesda, USA), Demis Kia (UCL Genetics Institute; and Department of Molecular Neuroscience, UCL Institute of Neurology, London, UK), Fabrice Danjou (Institut du Cerveau et de la Moelle-épinière (ICM), Paris, France), Faraz Faghri (Laboratory of Neurogenetics, National Institute on Aging, Bethesda, USA; Department of Computer Science, University of Illinois at Urbana-Champaign, Urbana, IL, USA), Gavin Charlesworth (Department of Molecular Neuroscience, UCL Institute of Neurology, London, UK), J Raphael Gibbs (Laboratory of Neurogenetics, National Institute on Aging, Bethsda, MD, USA; and Department of Molecular Neuroscience, UCL Institute of Neurology, London, UK), Huw R Morris (National Hospital for Neurology and Neurosurgery, University College London, London, UK), Helene Plun-Favreau (Department of Molecular Neuroscience, UCL Institute of Neurology, London, UK), Dena G Hernandez (Laboratory of Neurogenetics, National Institute on Aging, Bethesda, MD, USA; and Department of Molecular Neuroscience, UCL Institute of Neurology, London, UK), Peter Holmans (Biostatistics & Bioinformatics Unit, Institute of Psychological Medicine and Clinical Neuroscience, MRC Centre for Neuropsychiatric Genetics & Genomics, Cardiff, UK), Huw R Morris (National Hospital for Neurology and Neurosurgery, University College London, London, UK), Iris Jansen (VU University Medical Center, Amsterdam, Netherlands), John Hardy (Department of Molecular Neuroscience, UCL Institute of Neurology, London, UK), Javier Simón-Sánchez (Department for Neurodegenerative Diseases, Hertie Institute for Clinical Brain Research, University of Tübingen, and DZNE, German Center for Neurodegenerative Diseases, Tübingen, Germany), Jose M Bras (Department of Molecular Neuroscience, UCL Institute of Neurology, London, UK), Joshua Shulman (Baylor College of Medicine, Houston, Texas, USA), John Quinn (Institute of Translational Medicine, University of Liverpool, Liverpool, UK), Juan A. Botía (Universidad de Murcia, Murcia, Spain), Kin Y Mok (Department of Molecular Neuroscience, UCL Institute of Neurology, London, UK), Kimberley Billingsley (Institute of Translational Medicine, University of Liverpool, Liverpool, UK), Lasse Pihlstrom (Department of Neurology, Oslo University Hospital, Oslo, Norway), Lea R’Bibo (Department of Molecular Neuroscience, UCL Institute of Neurology, London, UK), Codrin Lungu (National Institutes of Health Parkinson Clinic, NINDS, National Institutes of Health, Bethesda, MD, USA), Manu Sharma (Centre for Genetic Epidemiology, Institute for Clinical Epidemiology and Applied Biometry, University of Tubingen and Department for Neurodegenerative Diseases, Hertie Institute for Clinical Brain Research, University of Tübingen Germany), Maria Martinez (INSERM UMR 1043; and Paul Sabatier University, Toulouse, France), Mina Ryten (Department of Molecular Neuroscience, UCL Institute of Neurology, London, UK), Valentina Escott-Price (MRC Centre for Neuropsychiatric Genetics and Genomics, Cardiff University School of Medicine, Cardiff, UK), Niccolo E. Mencacci (Department of Molecular Neuroscience, UCL, London, UK), Mike A. Nalls (Laboratory of Neurogenetics, National Institute on Aging, Bethesda, USA; Contractor/consultant with Kelly Services, Rockville, MD, USA), Nicholas W Wood (UCL Genetics Institute; and Department of Molecular Neuroscience, UCL Institute of Neurology, London, UK), Patrick Lewis (University of Reading, Reading, UK), Paul Denny (University College London, London, UK), Peter Heutink (DZNE, German Center for Neurodegenerative Diseases and Department for Neurodegenerative Diseases, Hertie Institute for Clinical Brain Research, University of Tübingen, Tübingen, Germany), Pille Taba (Department of Neurology and Neurosurgery, University of Tartu, Tartu, Estonia), Rita Guerreiro (Department of Molecular Neuroscience, UCL Institute of Neurology, London, UK), Ruth Lovering (University College London, London, UK), Raquel Duran Ogalla (University College London, London, UK), Rebecca Foulger (University College London, London, UK), Laurie Robak (Baylor College of Medicine, Houston, Texas, USA), Steven Lubbe (Ken and Ruth Davee Department of Neurology, Northwestern University Feinberg School of Medicine, Chicago, IL, USA), Steven Finkbeiner (Departments of Neurology and Physiology, University of California, San Francisco; Gladstone Institute of Neurological Disease; Taube/Koret Center for Neurodegenerative Disease Research, San Francisco, CA, USA), Shushant Jain (DZNE, German Center for Neurodegenerative Diseases and Department for Neurodegenerative Diseases, Hertie Institute for Clinical Brain Research, University of Tübingen, Tübingen, Germany), Sigurlaug Sveinbjörnsdóttir (Department of Neurology, Landspítali University Hospital, Reykjavík, Iceland; Department of Neurology, MEHT Broomfield Hospital, Chelmsford, Essex, UK; and Queen Mary College, University of London, London, UK), Andrew B Singleton (Laboratory of Neurogenetics, National Institute on Aging, Bethesda, MD, USA), Sonja Scholz (Neurodegenerative Diseases Research Unit, National Institute of Neurological Disorders and Stroke, Bethesda, MD, USA), Sulev Koks (Department of Neurology and Neurosurgery, University of Tartu, Tartu, Estonia), Suzanne Lesage (INSERM, UMR_S975 [ formerly UMR_S679], Paris, France; Université Pierre et Marie Curie-Paris, Centre de Recherche de l’Institut du Cerveau et de la Moelle épinière, Paris, France; and CNRS, Paris, France), Thomas Foltynie (UCL Institute of Neurology, London, UK), Thomas Gasser (Department for Neurodegenerative Diseases, Hertie Institute for Clinical Brain Research, and DZNE, German Center for Neurodegenerative Diseases, Tübingen, Germany), T. Ryan Price (University California Irvine, Irvine, CA, USA), Una-Marie Sheerin (Department of Molecular Neuroscience, UCL Institute of Neurology, London, UK), Nigel Williams (MRC Centre for Neuropsychiatric Genetics and Genomics, Cardiff, UK), Xylena Reed (Laboratory of Neurogenetics, National Institute on Aging, Bethesda, MD, USA), Zoltan Bochdanovits (Department of Clinical Genetics, Section of Medical Genomics, VU University Medical Centre, Amsterdam, Netherlands).

**Clumping of BMI variants**

Clumping was undertaken using the summary statistics files from the GIANT consortium website (<https://www.broadinstitute.org/collaboration/giant/index.php/Main_Page>). We used the combined sex, European ancestry data file.

Clumping generates a set of index SNPs that were independent of each other and associated with BMI at the genome wide significance level (i.e. 5x10^-8^). Index SNPs were identified by ranking associations with BMI from the smallest to largest p-value (but still with a cut-off value of p=5x10^-8^). Clumped SNPs were those in LD with index SNPs (R^2^ threshold of 0.001) or within 10,000 kilobases physical distance, when compared to a reference dataset (1000 Genomes Project <http://www.1000genomes.org/>). Hence each index SNP represented a number of clumped SNPs that were all associated with or near to the index SNP, and the index SNPs were all independent of one another (according to the parameters defined here). Independence of index SNPs is important because bias can be introduced if there is LD between them and can result to over-precise estimates in subsequent analysis. Standard code for clumping is available on the PLINK website (<http://pngu.mgh.harvard.edu/~purcell/plink/clump.shtml>).

The clumping parameters were as follows:

| Significance thresholds for index SNPs (p-value) | 5e^-8^ |
| --- | --- |
| Secondary significance threshold for clumped SNPs (p-value) | 5e^-8^ |
| LD threshold for clumping (R^2^) | 0·001 |
| Physical distance threshold for clumping (kilobases) | 10000 |

**S1 Table: Summarised tables of SNPs, nearest gene and putative action.**

| **Number of variants** | **SNPs (Genes)** | **Function of nearest gene** |
| --- | --- | --- |
| 9 | rs1516725 (ETV5), rs17405819 (HNF4G), rs6804842 (RARB), rs4740619 (CCDC171), rs9400239 (FOXO3), rs10733682 (LMX1B), rs17203016 (CREB1), rs2836754 (ETS2), rs977747 (NLRC3) | Transcription factor/regulation |
| 8 | rs2365389 (FHIT), rs12940622 (RPTOR), rs13191362 (PARK2), rs17001654 (SCARB2), rs6567160 (MC4R), rs11727676 (HHIP), rs6465468 (ASB4), rs1460676 (FIGN) | Protein or enzyme binding |
| 6 | rs17724992 (PGPEP1), rs1528435 (UBE2E3), rs657452 (AGBL4), rs3849570 (GBE1), rs2176598 (HSD17B12), rs492400 (USP37) | Enzyme activity |
| 6 | rs11165643 (PTBP2), rs7903146 (TCF7L2), rs11057405 (CLIP1), rs12401738 (FUBP1), rs2033732 (RALYL), rs6091540 (ZFP64) | Nucleic acid binding, DNA binding and RNA binding |
| 5 | rs9540493 (MIR548X2), rs9374842 (LOC285762), rs2176040 (LOC646736), rs1441264 (MIR548A2), rs2033529 (TDRG1) | RNA gene |
| 5 | rs12429545 (OLFM4), rs4256980 (TRIM66), rs17094222 (HIF1AN), rs7599312 (ERB4), rs1000940 (RABEP1) | Protein homodimerization |
| 4 | rs3101336 (NEGR1), rs7141420 (NRXN3), rs12286929 (CADM1), rs13078960 (CADM2) | Cell adhesion |
| 4 | rs7138803 (BCDIN3D), rs2287019 (QPCTL), rs16951275 (MAP2K5), rs7715256 (GALNT10) | Transferase (*various*) activity |
| 3 | rs1558902 (FTO), rs10938397 (GNPDA2), rs2207139 (TFAP2B) | Glucose and energy metabolism |
| 3 | rs17024393 (GNAT2), rs10132280 (STXBP6), rs16851483 (RASA2) | GTP binding and/or activity and/or signal transducer activity |
| 3 | rs10182181 (ADCY3), rs3888190 (ATP2A1), rs2121279 (LRP1B) | Nucleotide and/or calcium binding |
| 2 | rs11030104 (BDNF), rs1928295 (TLR4) | Cell signalling and receptor binding |
| 2 | rs1167827 (HIP1), rs6477694 (EPB41L4B) | Structural constituent of cytoskeleton |
| 1 | rs4787491 (INO80E) | DNA repair |
| 1 | rs13201877 (IFNGR1) | Cytokine receptor activity |
| 1 | rs543874 (SEC16B) | Golgi and vesicle transport |
| 1 | rs13107325 (SLC39A8) | Transmembrane transporter activity |
| 1 | rs758747 (TAL1) | Toll-like receptor signalling |
| 1 | rs3736485 (DMXL2) | GTPase binding |
| 1 | rs205262 (C6orf106) | Ubiquitin binding |
| 1 | rs7899106 (GRID1) | Glutamate receptor activity |
| 1 | rs29941 (KCTD15) | Intra-cellular signalling |
| 1 | rs887912 | Intergenic |
| 7 | rs13021737 (TMEM18), rs3817334 (MTCH2), rs2112347 (POC5), rs10968576 (LINGO2), rs1808579 (C18orf8), rs2820292 (NAV1), rs7239883 (LOC284260) | No further details |

Legend: Variants are listed here with the nearest genes and putative function of that gene listed by <http://www.genecards.org/>. For simplicity, only one possible function of each variant/gene is provided. Furthermore, just because a gene is near to a given variant, it does not mean that the variant exerts its effect on PD via BMI because of that gene.

**S2 Table: Full table of SNPs, locations, nearest genes and further information.**

| **SNP** | **Variance** | **Gene** | **Chrom-osome** | **BP** | **Genecard description** (<http://www.genecards.org/>) |
| --- | --- | --- | --- | --- | --- |
| rs1558902 | 0·32% | FTO | 16 | 52361075 | FTO (Fat Mass And Obesity Associated) is a Protein Coding gene. Diseases associated with FTO include lethal polymalformative syndrome, boissel type and growth retardation, developmental delay, coarse facies, and early death. Among its related pathways are Glucose / Energy Metabolism. GO annotations related to this gene include ferrous iron binding and oxidative RNA demethylase activity. |
| rs6567160 | 0·11% | MC4R | 18 | 55980115 | MC4R (Melanocortin 4 Receptor) is a Protein Coding gene. Diseases associated with MC4R include obesity and obesity due to melanocortin 4 receptor deficiency. Among its related pathways are signaling by GPCR and Peptide ligand-binding receptors. GO annotations related to this gene include G-protein coupled receptor activity and peptide hormone binding. An important paralog of this gene is LPAR1. |
| rs13021737 | 0·10% | TMEM18 | 2 | 622348 | TMEM18 (Transmembrane Protein 18) is a Protein Coding gene. |
| rs10938397 | 0·08% | GNPDA2 | 4 | 44877284 | GNPDA2 (Glucosamine-6-Phosphate Deaminase 2) is a Protein Coding gene. Diseases associated with GNPDA2 include obesity. Among its related pathways are Metabolism and Amino sugar and nucleotide sugar metabolism. GO annotations related to this gene include hydrolase activity and glucosamine-6-phosphate deaminase activity. An important paralog of this gene is GNPDA1. |
| rs543874 | 0·08% | SEC16B | 1 | 176156103 | SEC16B (SEC16 Homolog B, Endoplasmic Reticulum Export Factor) is a Protein Coding gene. Among its related pathways are Transport to the Golgi and subsequent modification and Vesicle-mediated transport. An important paralog of this gene is SEC16A. |
| rs11030104 | 0·06% | BDNF | 11 | 27641093 | BDNF (Brain-Derived Neurotrophic Factor) is a Protein Coding gene. Diseases associated with BDNF include anorexia nervosa and bulimia nervosa, age of onset of weight loss in. Among its related pathways are MAPK signaling pathway and NF-Kappa B Family Pathway. GO annotations related to this gene include receptor binding and neurotrophin TRKB receptor binding. An important paralog of this gene is NTF3. |
| rs2207139 | 0·06% | TFAP2B | 6 | 50953449 | TFAP2B (Transcription Factor AP-2 Beta (Activating Enhancer Binding Protein 2 Beta)) is a Protein Coding gene. Diseases associated with TFAP2B include char syndrome and patent ductus arteriosus. Among its related pathways are Neural Crest Differentiation and Glucose / Energy Metabolism. GO annotations related to this gene include transcription factor activity, sequence-specific DNA binding and sequence-specific DNA binding. An important paralog of this gene is TFAP2E. |
| rs10182181 | 0·05% | ADCY3 | 2 | 25003800 | ADCY3 (Adenylate Cyclase 3) is a Protein Coding gene. Among its related pathways are Immune System and DAG and IP3 signaling. GO annotations related to this gene include nucleotide binding and phosphorus-oxygen lyase activity. An important paralog of this gene is ADCY5. |
| rs3888190 | 0·05% | ATP2A1 | 16 | 28796987 | ATP2A1 (ATPase, Ca++ Transporting, Cardiac Muscle, Fast Twitch 1) is a Protein Coding gene. Diseases associated with ATP2A1 include brody myopathy and pseudomyotonia. Among its related pathways are signaling by GPCR and CREB Pathway. GO annotations related to this gene include calcium ion binding and nucleotide binding. An important paralog of this gene is ATP2A2. |
| rs7138803 | 0·05% | BCDIN3D | 12 | 48533735 | BCDIN3D (BCDIN3 Domain Containing) is a Protein Coding gene. GO annotations related to this gene include methyltransferase activity and RNA methyltransferase activity. |
| rs1516725 | 0·05% | ETV5 | 3 | 187306698 | ETV5 (Ets Variant 5) is a Protein Coding gene. Diseases associated with ETV5 include sertoli cell-only syndrome. Among its related pathways are Transcriptional misregulation in cancer and Androgen receptor signaling pathway. GO annotations related to this gene include transcription factor activity, sequence-specific DNA binding and transcription regulatory region DNA binding. An important paralog of this gene is ETV2. |
| rs3101336 | 0·05% | NEGR1 | 1 | 72523773 | NEGR1 (Neuronal Growth Regulator 1) is a Protein Coding gene. Diseases associated with NEGR1 include podoconiosis. Among its related pathways are Cell adhesion molecules (CAMs). An important paralog of this gene is NTM. |
| rs2287019 | 0·04% | QPCTL | 19 | 50894012 | QPCTL (Glutaminyl-Peptide Cyclotransferase-Like) is a Protein Coding gene. GO annotations related to this gene include glutaminyl-peptide cyclotransferase activity. An important paralog of this gene is QPCT. |
| rs657452 | 0·03% | AGBL4 | 1 | 49362434 | AGBL4 (ATP/GTP Binding Protein-Like 4) is a Protein Coding gene. Diseases associated with AGBL4 include macular degeneration, age-related, 1. GO annotations related to this gene include tubulin binding and metallocarboxypeptidase activity. An important paralog of this gene is AGBL2. |
| rs13078960 | 0·03% | CADM2 | 3 | 85890280 | CADM2 (Cell Adhesion Molecule 2) is a Protein Coding gene. Among its related pathways are cell junction organization. An important paralog of this gene is CADM3. |
| rs17024393 | 0·03% | GNAT2 | 1 | 109956211 | GNAT2 (Guanine Nucleotide Binding Protein (G Protein), Alpha Transducing Activity Polypeptide 2) is a Protein Coding gene. Diseases associated with GNAT2 include achromatopsia-4 and achromatopsia. Among its related pathways are signaling by GPCR and Apoptotic Pathways in Synovial Fibroblasts. GO annotations related to this gene include GTP binding and signal transducer activity. An important paralog of this gene is GNAQ. |
| rs10968576 | 0·03% | LINGO2 | 9 | 28404339 | LINGO2 (Leucine Rich Repeat And Ig Domain Containing 2) is a Protein Coding gene. Diseases associated with LINGO2 include essential tremor. An important paralog of this gene is LINGO1. |
| rs16951275 | 0·03% | MAP2K5 | 15 | 65864222 | MAP2K5 (Mitogen-Activated Protein Kinase Kinase 5) is a Protein Coding gene. Diseases associated with MAP2K5 include caroli disease and restless legs syndrome. Among its related pathways are MAPK signaling pathway and Signaling by GPCR. GO annotations related to this gene include transferase activity, transferring phosphorus-containing groups and protein tyrosine kinase activity. An important paralog of this gene is MAP2K7. |
| rs3817334 | 0·03% | MTCH2 | 11 | 47607569 | MTCH2 (Mitochondrial Carrier 2) is a Protein Coding gene. Diseases associated with MTCH2 include obesity. An important paralog of this gene is MTCH1. |
| rs7141420 | 0·03% | NRXN3 | 14 | 78969207 | NRXN3 (Neurexin 3) is a Protein Coding gene. Diseases associated with NRXN3 include autism spectrum disorder and borderline personality disorder. Among its related pathways are Muscular Dystrophies and Dystrophin-Glycoprotein Complex and Cell adhesion molecules (CAMs). GO annotations related to this gene include receptor activity and cell adhesion molecule binding. An important paralog of this gene is CNTNAP1. |
| rs12429545 | 0·03% | OLFM4 | 13 | 53000207 | OLFM4 (Olfactomedin 4) is a Protein Coding gene. Diseases associated with OLFM4 include epileptic encephalopathy, early infantile, 3 and pancreatic cancer. Among its related pathways are Adhesion and DNA Damage. GO annotations related to this gene include protein homodimerization activity and cadherin binding. An important paralog of this gene is GLDN. |
| rs2112347 | 0·03% | POC5 | 5 | 75050998 | POC5 (POC5 Centriolar Protein) is a Protein Coding gene. |
| rs16851483 | 0·03% | RASA2 | 3 | 142758126 | RASA2 (RAS P21 Protein Activator 2) is a Protein Coding gene. Diseases associated with RASA2 include kuru and neurofibromatosis, type 1. Among its related pathways are MAPK signaling pathway and Immune System. GO annotations related to this gene include GTPase activator activity. An important paralog of this gene is RASAL1. |
| rs13107325 | 0·03% | SLC39A8 | 4 | 103407732 | SLC39A8 (Solute Carrier Family 39 (Zinc Transporter), Member 8) is a Protein Coding gene. Among its related pathways are Transport of glucose and other sugars, bile salts and organic acids, metal ions and amine compounds and Metal ion SLC transporters. GO annotations related to this gene include metal ion transmembrane transporter activity. An important paralog of this gene is SLC39A12. |
| rs205262 | 0·02% | C6orf106 | 6 | 34671142 | C6orf106 (Chromosome 6 Open Reading Frame 106) is a Protein Coding gene. GO annotations related to this gene include ubiquitin binding. |
| rs12286929 | 0·02% | CADM1 | 11 | 114527614 | CADM1 (Cell Adhesion Molecule 1) is a Protein Coding gene. Diseases associated with CADM1 include retroperitoneal fibrosis and asthenopia. Among its related pathways are Cell junction organization and Cell adhesion molecules (CAMs). GO annotations related to this gene include protein homodimerization activity and PDZ domain binding. An important paralog of this gene is CADM3. |
| rs11057405 | 0·02% | CLIP1 | 12 | 121347850 | CLIP1 (CAP-GLY Domain Containing Linker Protein 1) is a Protein Coding gene. Diseases associated with CLIP1 include extratemporal epilepsy and distal hereditary motor neuropathy, type ii. Among its related pathways are signaling by GPCR and PAK Pathway. GO annotations related to this gene include nucleic acid binding and microtubule binding. An important paralog of this gene is CLIP4. |
| rs7599312 | 0·02% | ERBB4 | 2 | 213121476 | ERBB4 (Erb-B2 Receptor Tyrosine Kinase 4) is a Protein Coding gene. Diseases associated with ERBB4 include amyotrophic lateral sclerosis 19 and erbb4-related amyotrophic lateral sclerosis. Among its related pathways are PI3K/AKT signaling in Cancer and Immune System. GO annotations related to this gene include protein homodimerization activity and protein kinase activity. An important paralog of this gene is TNK1. |
| rs2365389 | 0·02% | FHIT | 3 | 61211502 | FHIT (Fragile Histidine Triad) is a Protein Coding gene. Diseases associated with FHIT include hereditary conventional renal cell carcinoma and hereditary clear cell renal cell carcinoma. Among its related pathways are Glioma and Small cell lung cancer. GO annotations related to this gene include identical protein binding and hydrolase activity. |
| rs12401738 | 0·02% | FUBP1 | 1 | 78219349 | FUBP1 (Far Upstream Element Binding Protein 1) is a Protein Coding gene. Diseases associated with FUBP1 include oligodendroglioma and mouth disease. GO annotations related to this gene include nucleic acid binding and transcription factor activity, sequence-specific DNA binding. An important paralog of this gene is FUBP3. |
| rs3849570 | 0·02% | GBE1 | 3 | 81874802 | GBE1 (Glucan (1,4-Alpha-), Branching Enzyme 1) is a Protein Coding gene. Diseases associated with GBE1 include glycogen storage disease iv and polyglucosan body disease, adult form. Among its related pathways are Metabolism and Glycosaminoglycan metabolism. GO annotations related to this gene include hydrolase activity, hydrolyzing O-glycosyl compounds and 1,4-alpha-glucan branching enzyme activity. |
| rs7899106 | 0·02% | GRID1 | 10 | 87400884 | GRID1 (Glutamate Receptor, Ionotropic, Delta 1) is a Protein Coding gene. Diseases associated with GRID1 include bipolar disorder and schizophrenia. Among its related pathways are Peptide ligand-binding receptors. GO annotations related to this gene include ionotropic glutamate receptor activity and extracellular-glutamate-gated ion channel activity. An important paralog of this gene is GRID2. |
| rs11727676 | 0·02% | HHIP | 4 | 145878514 | HHIP (Hedgehog Interacting Protein) is a Protein Coding gene. Diseases associated with HHIP include basal cell carcinoma and holoprosencephaly. Among its related pathways are signaling by GPCR and Pathways in cancer. GO annotations related to this gene include quinone binding and hedgehog family protein binding. An important paralog of this gene is HHIPL2. |
| rs17094222 | 0·02% | HIF1AN | 10 | 102385430 | HIF1AN (Hypoxia Inducible Factor 1, Alpha Subunit Inhibitor) is a Protein Coding gene. Diseases associated with HIF1AN include hypoxia and severe pre-eclampsia. Among its related pathways are Angiogenesis (CST) and Cellular Senescence. GO annotations related to this gene include protein homodimerization activity and oxidoreductase activity, acting on paired donors, with incorporation or reduction of molecular oxygen, 2-oxoglutarate as one donor, and incorporation of one atom each of oxygen into both donors. An important paralog of this gene is HSPBAP1. |
| rs1167827 | 0·02% | HIP1 | 7 | 75001105 | HIP1 (Huntingtin Interacting Protein 1) is a Protein Coding gene. Diseases associated with HIP1 include huntington disease and chronic myelomonocytic leukemia. Among its related pathways are fMLP Pathway and Coregulation of Androgen receptor activity. GO annotations related to this gene include binding and structural constituent of cytoskeleton. An important paralog of this gene is TLN1. |
| rs17405819 | 0·02% | HNF4G | 8 | 76969139 | HNF4G (Hepatocyte Nuclear Factor 4, Gamma) is a Protein Coding gene. Diseases associated with HNF4G include maturity-onset diabetes of the young. Among its related pathways are Developmental Biology and Regulation of beta-cell development. GO annotations related to this gene include transcription factor activity, sequence-specific DNA binding and steroid hormone receptor activity. An important paralog of this gene is RXRG. |
| rs10733682 | 0·02% | LMX1B | 9 | 128500735 | LMX1B (LIM Homeobox Transcription Factor 1 Beta) is a Protein Coding gene. Diseases associated with LMX1B include nail-patella syndrome and genitopatellar syndrome. Among its related pathways are SIDS Susceptibility Pathways. GO annotations related to this gene include transcription factor activity, sequence-specific DNA binding and sequence-specific DNA binding. An important paralog of this gene is LHX4. |
| rs2121279 | 0·02% | LRP1B | 2 | 142759755 | LRP1B (Low Density Lipoprotein Receptor-Related Protein 1B) is a Protein Coding gene. Diseases associated with LRP1B include endocervical carcinoma. GO annotations related to this gene include calcium ion binding and low-density lipoprotein receptor activity. An important paralog of this gene is LDLR. |
| rs9540493 | 0·02% | MIR548X2 | 13 | 65103705 | MIR548X2 (MicroRNA 548x-2) is an RNA Gene, and is affiliated with the miRNA class. |
| rs2820292 | 0·02% | NAV1 | 1 | 200050910 | NAV1 (Neuron Navigator 1) is a Protein Coding gene. Diseases associated with NAV1 include myotonia congenita, dominant and brugada syndrome. An important paralog of this gene is NAV3. |
| rs758747 | 0·02% | NLRC3 | 16 | 3567359 | NLRC3 (NLR Family, CARD Domain Containing 3) is a Protein Coding gene. Among its related pathways are Toll-Like receptor Signaling Pathways. An important paralog of this gene is NLRP7. |
| rs13191362 | 0·02% | PARK2 | 6 | 162953340 | PARK2 (Parkin RBR E3 Ubiquitin Protein Ligase) is a Protein Coding gene. Diseases associated with PARK2 include young-onset parkinson disease and parkinson disease, juvenile, type 2. Among its related pathways are Immune System and Alpha-synuclein signaling. GO annotations related to this gene include identical protein binding and enzyme binding. |
| rs17724992 | 0·02% | PGPEP1 | 19 | 18315825 | PGPEP1 (Pyroglutamyl-Peptidase I) is a Protein Coding gene. Among its related pathways are Carbon metabolism and Metabolism. GO annotations related to this gene include cysteine-type peptidase activity and pyroglutamyl-peptidase activity. An important paralog of this gene is PGPEP1L. |
| rs11165643 | 0·02% | PTBP2 | 1 | 96696685 | PTBP2 (Polypyrimidine Tract Binding Protein 2) is a Protein Coding gene. Diseases associated with PTBP2 include cancer-associated retinopathy. Among its related pathways are mRNA Splicing - Major Pathway. GO annotations related to this gene include nucleic acid binding and RNA binding. An important paralog of this gene is ENSG00000268083. |
| rs1000940 | 0·02% | RABEP1 | 17 | 5223976 | RABEP1 (Rabaptin, RAB GTPase Binding Effector Protein 1) is a Protein Coding gene. Among its related pathways are Endocytosis and wtCFTR and delta508-CFTR traffic / Generic schema (norm and CF). GO annotations related to this gene include protein homodimerization activity and growth factor activity. An important paralog of this gene is RABEP2. |
| rs6804842 | 0·02% | RARB | 3 | 25081441 | RARB (Retinoic Acid Receptor, Beta) is a Protein Coding gene. Diseases associated with RARB include microphthalmia, syndromic 12 and chromosome 3p deletion. Among its related pathways are signaling by GPCR and Pathways in cancer. GO annotations related to this gene include transcription factor activity, sequence-specific DNA binding and protein complex binding. An important paralog of this gene is NR1I2. |
| rs12940622 | 0·02% | RPTOR | 17 | 76230166 | RPTOR (Regulatory Associated Protein Of MTOR, Complex 1) is a Protein Coding gene. Diseases associated with RPTOR include aqueous misdirection and rubeosis iridis. Among its related pathways are PI3K-Akt signaling pathway and signaling by GPCR. GO annotations related to this gene include binding and protein complex binding. |
| rs17001654 | 0·02% | SCARB2 | 4 | 77348592 | SCARB2 (Scavenger Receptor Class B, Member 2) is a Protein Coding gene. Diseases associated with SCARB2 include epilepsy, progressive myoclonic 4, with or without renal failure and hand, foot and mouth disease. Among its related pathways are Lysosome. GO annotations related to this gene include enzyme binding and receptor activity. An important paralog of this gene is CD36. |
| rs10132280 | 0·02% | STXBP6 | 14 | 24998019 | STXBP6 (Syntaxin Binding Protein 6 (Amisyn)) is a Protein Coding gene. GO annotations related to this gene include phosphatidylinositol-4,5-bisphosphate binding and GTP-Rho binding. An important paralog of this gene is EXOC1. |
| rs7903146 | 0·02% | TCF7L2 | 10 | 114748339 | TCF7L2 (Transcription Factor 7-Like 2 (T-Cell Specific, HMG-Box)) is a Protein Coding gene. Diseases associated with TCF7L2 include tropical calcific pancreatitis and diabetes mellitus, noninsulin-dependent. Among its related pathways are signaling by GPCR and Downstream signaling events of B Cell Receptor (BCR). GO annotations related to this gene include transcription factor activity, sequence-specific DNA binding and chromatin binding. An important paralog of this gene is TCF7. |
| rs1928295 | 0·02% | TLR4 | 9 | 119418304 | TLR4 (Toll-Like Receptor 4) is a Protein Coding gene. Diseases associated with TLR4 include macular degeneration, age-related, 10 and pertussis. Among its related pathways are PI3K-Akt signaling pathway and Immune System. GO annotations related to this gene include receptor activity and lipopolysaccharide binding. An important paralog of this gene is TLR6. |
| rs4256980 | 0·02% | TRIM66 | 11 | 8630515 | TRIM66 (Tripartite Motif Containing 66) is a Protein Coding gene. GO annotations related to this gene include protein homodimerization activity and chromatin binding. An important paralog of this gene is TRIM28. |
| rs6465468 | 0·01% | ASB4 | 7 | 95007450 | ASB4 (Ankyrin Repeat And SOCS Box Containing 4) is a Protein Coding gene. Among its related pathways are Immune System and Antigen processing- Ubiquitination and Proteasome degradation. GO annotations related to this gene include enzyme binding and ubiquitin protein ligase binding. An important paralog of this gene is ASB10. |
| rs1808579 | 0·01% | C18orf8 | 18 | 19358886 | C18orf8 (Chromosome 18 Open Reading Frame 8) is a Protein Coding gene. |
| rs4740619 | 0·01% | CCDC171 | 9 | 15624326 | CCDC171 (Coiled-Coil Domain Containing 171) is a Protein Coding gene. GO annotations related to this gene include transcription factor activity, sequence-specific DNA binding and signal transducer activity. |
| rs17203016 | 0·01% | CREB1 | 2 | 207963763 | CREB1 (CAMP Responsive Element Binding Protein 1) is a Protein Coding gene. Diseases associated with CREB1 include histiocytoma, angiomatoid fibrous, somatic and melanoma of soft parts. Among its related pathways are signalling by GPCR and immune System. GO annotations related to this gene include transcription factor activity, sequence-specific DNA binding and enzyme binding. An important paralog of this gene is ATF1. |
| rs3736485 | 0·01% | DMXL2 | 15 | 49535902 | DMXL2 (Dmx-Like 2) is a Protein Coding gene. Diseases associated with DMXL2 include polyendocrine-polyneuropathy syndrome and hypertrophy of breast. GO annotations related to this gene include Rab GTPase binding. An important paralog of this gene is DMXL1. |
| rs6477694 | 0·01% | EPB41L4B | 9 | 110972163 | EPB41L4B (Erythrocyte Membrane Protein Band 4.1 Like 4B) is a Protein Coding gene. GO annotations related to this gene include structural constituent of cytoskeleton and cytoskeletal protein binding. An important paralog of this gene is FRMD3. |
| rs2836754 | 0·01% | ETS2 | 21 | 39213610 | ETS2 (ETS Proto-Oncogene 2, Transcription Factor) is a Protein Coding gene. Diseases associated with ETS2 include choriocarcinoma and down syndrome. Among its related pathways are Cellular Senescence and Ras signaling pathway. GO annotations related to this gene include transcription factor activity, sequence-specific DNA binding and RNA polymerase II core promoter proximal region sequence-specific DNA binding. An important paralog of this gene is ETV2. |
| rs1460676 | 0·01% | FIGN | 2 | 164275935 | FIGN (Fidgetin) is a Protein Coding gene. GO annotations related to this gene include protein C-terminus binding and microtubule-severing ATPase activity. An important paralog of this gene is IQCA1L. |
| rs9400239 | 0·01% | FOXO3 | 6 | 109084356 | FOXO3 (Forkhead Box O3) is a Protein Coding gene. Diseases associated with FOXO3 include rhabdomyosarcoma and lung cancer. Among its related pathways are PI3K-Akt signaling pathway and PI3K/AKT Signaling in Cancer. GO annotations related to this gene include transcription factor activity, sequence-specific DNA binding and protein kinase binding. An important paralog of this gene is FOXO4. |
| rs7715256 | 0·01% | GALNT10 | 5 | 153518086 | GALNT10 (Polypeptide N-Acetylgalactosaminyltransferase 10) is a Protein Coding gene. Among its related pathways are Metabolism and O-linked glycosylation. GO annotations related to this gene include carbohydrate binding and polypeptide N-acetylgalactosaminyltransferase activity. An important paralog of this gene is GALNT4. |
| rs2176598 | 0·01% | HSD17B12 | 11 | 43820854 | HSD17B12 (Hydroxysteroid (17-Beta) Dehydrogenase 12) is a Protein Coding gene. Among its related pathways are Metabolism and Regulation of lipid metabolism by Peroxisome proliferator-activated receptor alpha (PPARalpha). GO annotations related to this gene include oxidoreductase activity and collagen binding. An important paralog of this gene is HSDL1. |
| rs13201877 | 0·01% | IFNGR1 | 6 | 137717234 | IFNGR1 (Interferon Gamma Receptor 1) is a Protein Coding gene. Diseases associated with IFNGR1 include immunodeficiency 27a, mycobacteriosis, ar and immunodeficiency 27b, mycobacteriosis, ad. Among its related pathways are immune system and interferon gamma signalling. GO annotations related to this gene include cytokine receptor activity and interferon-gamma receptor activity. |
| rs4787491 | 0·01% | INO80E | 16 | 29922838 | INO80E (INO80 Complex Subunit E) is a Protein Coding gene. Among its related pathways are DNA Double-Strand Break Repair and Transcription-Coupled Nucleotide Excision Repair (TC-NER). |
| rs29941 | 0·01% | KCTD15 | 19 | 39001372 | KCTD15 (Potassium Channel Tetramerization Domain Containing 15) is a Protein Coding gene. Among its related pathways are Activation of cAMP-Dependent PKA and Neuropathic Pain-Signaling in Dorsal Horn Neurons. An important paralog of this gene is KCTD11. |
| rs7239883 | 0·01% | LOC284260 | 18 | 38401669 | No additional information |
| rs9374842 | 0·01% | LOC285762 | 6 | 120227364 | LOC285762 (Uncharacterized LOC285762) is an RNA Gene, and is affiliated with the ncRNA class. |
| rs2176040 | 0·01% | LOC646736 | 2 | 226801046 | LOC646736 (Uncharacterized LOC646736) is an RNA Gene, and is affiliated with the ncRNA class. |
| rs1441264 | 0·01% | MIR548A2 | 13 | 78478920 | MIR548A2 (MicroRNA 548a-2) is an RNA Gene, and is affiliated with the miRNA class. |
| rs2033732 | 0·01% | RALYL | 8 | 85242264 | RALYL (RALY RNA Binding Protein-Like) is a Protein Coding gene. GO annotations related to this gene include nucleic acid binding and identical protein binding. An important paralog of this gene is HNRNPCL3. |
| rs977747 | 0·01% | TAL1 | 1 | 47457264 | TAL1 (T-Cell Acute Lymphocytic Leukemia 1) is a Protein Coding gene. Diseases associated with TAL1 include leukemia, acute lymphoblastic and precursor t-cell acute lymphoblastic leukemia. GO annotations related to this gene include transcription factor activity, sequence-specific DNA binding and chromatin binding. An important paralog of this gene is LYL1. |
| rs2033529 | 0·01% | TDRG1 | 6 | 40456631 | TDRG1 (Testis Development Related 1 (Non-Protein Coding)) is an RNA Gene, and is affiliated with the non-coding RNA class. |
| rs1528435 | 0·01% | UBE2E3 | 2 | 181259207 | UBE2E3 (Ubiquitin-Conjugating Enzyme E2E 3) is a Protein Coding gene. Among its related pathways are Immune System and Remodeling of Adherens Junctions. GO annotations related to this gene include ligase activity and acid-amino acid ligase activity. An important paralog of this gene is UBE2D3. |
| rs492400 | 0·01% | USP37 | 2 | 219057996 | USP37 (Ubiquitin Specific Peptidase 37) is a Protein Coding gene. Among its related pathways are Ubiquitin-Proteasome Dependent Proteolysis. GO annotations related to this gene include protein kinase binding and thiol-dependent ubiquitin-specific protease activity. An important paralog of this gene is USP29. |
| rs6091540 | 0·01% | ZFP64 | 20 | 50521269 | ZFP64 (ZFP64 Zinc Finger Protein) is a Protein Coding gene. GO annotations related to this gene include nucleic acid binding. An important paralog of this gene is RREB1. |
| rs887912 | 0·00% | Intergenic | 2 | 59075742 | Intergenic |

Legend: BP = base pair; GO = gene ontology; information relating to variance in BMI explained by each SNP comes from the supplementary files of Locke et al. Nature 2015 [17]

**S3 Table: Log odds ratios, standard errors and p-value for instrumental variable analysis.**

|  | **SNP** | **log- OR** | **SE** | **p-value** |  | **SNP** | **log- OR** | **SE** | **p-value** | **p-value for heterogeneity** |
| --- | --- | --- | --- | --- | --- | --- | --- | --- | --- | --- |
| 1 | rs17001654 | -2·55 | 0·80 | 0·001 | 64 | rs7239883 | 0·63 | 1·09 | 0·562 |  |
| 2 | rs13107325 | -2·31 | 0·68 | 0·001 | 65 | rs2836754 | 0·64 | 0·98 | 0·514 |  |
| 3 | rs4787491 | -2·09 | 1·07 | 0·051 | 66 | rs12286929 | 0·66 | 0·76 | 0·385 |  |
| 4 | rs4740619 | -1·73 | 0·98 | 0·078 | 67 | rs2176598 | 0·70 | 0·97 | 0·470 |  |
| 5 | rs2820292 | -1·70 | 0·90 | 0·059 | 68 | rs3736485 | 0·80 | 1·02 | 0·432 |  |
| 6 | rs1808579 | -1·69 | 1·00 | 0·090 | 69 | rs10733682 | 0·91 | 0·84 | 0·277 |  |
| 7 | rs3888190 | -1·44 | 0·54 | 0·008 | 70 | rs13191362 | 1·20 | 0·86 | 0·162 |  |
| 8 | rs2033732 | -1·28 | 1·07 | 0·233 | 71 | rs9374842 | 1·28 | 0·94 | 0·172 |  |
| 9 | rs6465468 | -1·26 | 1·17 | 0·282 | 72 | rs887912 | 1·38 | 0·77 | 0·073 |  |
| 10 | rs12401738 | -1·15 | 0·86 | 0·181 | 73 | rs9540493 | 1·41 | 0·95 | 0·139 |  |
| 11 | rs1000940 | -1·12 | 0·97 | 0·248 | 74 | rs977747 | 1·43 | 0·96 | 0·138 |  |
| 12 | rs6091540 | -1·05 | 0·94 | 0·266 | 75 | rs2121279 | 1·54 | 1·00 | 0·122 |  |
| 13 | rs11727676 | -1·01 | 1·14 | 0·374 | 76 | rs9400239 | 1·62 | 1·04 | 0·118 |  |
| 14 | rs16851483 | -0·96 | 0·69 | 0·165 | 77 | rs3849570 | 1·65 | 0·94 | 0·081 |  |
| 15 | rs10968576 | -0·96 | 0·69 | 0·167 |  |  |  |  |  |  |
| 16 | rs10132280 | -0·95 | 0·82 | 0·205 |  |  |  |  |  |  |
| 17 | rs492400 | -0·94 | 1·13 | 0·407 |  | **IVW effect** | **-0·19** | **0·09** | **0·029** | **0·065** |
| 18 | rs7899106 | -0·90 | 1·03 | 0·382 |  |  |  |  |  |  |
| 19 | rs1441264 | -0·87 | 0·96 | 0·364 |  |  |  |  |  |  |
| 20 | rs3101336 | -0·80 | 0·51 | 0·120 |  |  |  |  |  |  |
| 21 | rs13078960 | -0·77 | 0·69 | 0·262 |  |  |  |  |  |  |
| 22 | rs17094222 | -0·77 | 0·81 | 0·344 |  |  |  |  |  |  |
| 23 | rs11165643 | -0·72 | 0·74 | 0·329 |  |  |  |  |  |  |
| 24 | rs12940622 | -0·66 | 0·89 | 0·460 |  |  |  |  |  |  |
| 25 | rs11030104 | -0·64 | 0·47 | 0·178 |  |  |  |  |  |  |
| 26 | rs7599312 | -0·61 | 0·87 | 0·482 |  |  |  |  |  |  |
| 27 | rs6567160 | -0·54 | 0·34 | 0·115 |  |  |  |  |  |  |
| 28 | rs2176040 | -0·51 | 1·11 | 0·645 |  |  |  |  |  |  |
| 29 | rs1516725 | -0·49 | 0·52 | 0·347 |  |  |  |  |  |  |
| 30 | rs17724992 | -0·46 | 0·91 | 0·617 |  |  |  |  |  |  |
| 31 | rs7715256 | -0·45 | 0·96 | 0·637 |  |  |  |  |  |  |
| 32 | rs12429545 | -0·34 | 0·74 | 0·646 |  |  |  |  |  |  |
| 33 | rs1558902 | -0·27 | 0·20 | 0·187 |  |  |  |  |  |  |
| 34 | rs17203016 | -0·27 | 1·00 | 0·790 |  |  |  |  |  |  |
| 35 | rs657452 | -0·26 | 0·72 | 0·721 |  |  |  |  |  |  |
| 36 | rs1460676 | -0·24 | 1·06 | 0·822 |  |  |  |  |  |  |
| 37 | rs6804842 | -0·23 | 0·90 | 0·795 |  |  |  |  |  |  |
| 38 | rs4256980 | -0·22 | 0·80 | 0·785 |  |  |  |  |  |  |
| 39 | rs1528435 | -0·20 | 0·92 | 0·828 |  |  |  |  |  |  |
| 40 | rs16951275 | -0·20 | 0·64 | 0·757 |  |  |  |  |  |  |
| 41 | rs10182181 | -0·18 | 0·52 | 0·726 |  |  |  |  |  |  |
| 42 | rs17024393 | -0·17 | 0·80 | 0·832 |  |  |  |  |  |  |
| 43 | rs3817334 | -0·16 | 0·63 | 0·803 |  |  |  |  |  |  |
| 44 | rs1928295 | -0·14 | 0·89 | 0·876 |  |  |  |  |  |  |
| 45 | rs2112347 | -0·10 | 0·87 | 0·905 |  |  |  |  |  |  |
| 46 | rs2365389 | -0·03 | 0·82 | 0·966 |  |  |  |  |  |  |
| 47 | rs758747 | 0·00 | 0·80 | 1·000 |  |  |  |  |  |  |
| 48 | rs13201877 | 0·12 | 1·08 | 0·914 |  |  |  |  |  |  |
| 49 | rs7138803 | 0·16 | 0·52 | 0·759 |  |  |  |  |  |  |
| 50 | rs2287019 | 0·21 | 0·57 | 0·719 |  |  |  |  |  |  |
| 51 | rs2207139 | 0·24 | 0·47 | 0·606 |  |  |  |  |  |  |
| 52 | rs17405819 | 0·29 | 0·79 | 0·717 |  |  |  |  |  |  |
| 53 | rs10938397 | 0·34 | 0·41 | 0·410 |  |  |  |  |  |  |
| 54 | rs29941 | 0·35 | 0·95 | 0·713 |  |  |  |  |  |  |
| 55 | rs7141420 | 0·35 | 0·72 | 0·626 |  |  |  |  |  |  |
| 56 | rs2033529 | 0·36 | 0·98 | 0·718 |  |  |  |  |  |  |
| 57 | rs6477694 | 0·38 | 0·98 | 0·702 |  |  |  |  |  |  |
| 58 | rs13021737 | 0·38 | 0·35 | 0·273 |  |  |  |  |  |  |
| 59 | rs1167827 | 0·43 | 0·81 | 0·600 |  |  |  |  |  |  |
| 60 | rs7903146 | 0·46 | 0·73 | 0·530 |  |  |  |  |  |  |
| 61 | rs543874 | 0·49 | 0·41 | 0·226 |  |  |  |  |  |  |
| 62 | rs205262 | 0·51 | 0·84 | 0·545 |  |  |  |  |  |  |
| 63 | rs11057405 | 0·60 | 0·99 | 0·543 |  |  |  |  |  |  |

Legend: SNP = single nucleotide polymorphism; IVW = inverse weighted variance; SE = standard error; OR = odds ratio

**S4 Table: Leave one out analysis (inverse variance weighting method)**

|  | **Missing SNP** | **OR** | **LCI** | **UCI** | **p-value** |  | **Missing SNP** | **OR** | **LCI** | **UCI** | **p-value** |
| --- | --- | --- | --- | --- | --- | --- | --- | --- | --- | --- | --- |
| 1 | rs1000940 | 0·83 | 0·70 | 0·98 | 0·035 | 64 | rs6804842 | 0·83 | 0·70 | 0·98 | 0·031 |
| 2 | rs10132280 | 0·83 | 0·70 | 0·98 | 0·036 | 65 | rs7138803 | 0·82 | 0·69 | 0·97 | 0·025 |
| 3 | rs10182181 | 0·82 | 0·69 | 0·98 | 0·032 | 66 | rs7141420 | 0·82 | 0·69 | 0·97 | 0·026 |
| 4 | rs10733682 | 0·82 | 0·69 | 0·97 | 0·022 | 67 | rs7239883 | 0·82 | 0·69 | 0·97 | 0·027 |
| 5 | rs10938397 | 0·81 | 0·68 | 0·96 | 0·018 | 68 | rs758747 | 0·82 | 0·69 | 0·98 | 0·029 |
| 6 | rs10968576 | 0·83 | 0·70 | 0·99 | 0·039 | 69 | rs7599312 | 0·83 | 0·70 | 0·98 | 0·033 |
| 7 | rs11030104 | 0·83 | 0·70 | 0·99 | 0·043 | 70 | rs7715256 | 0·83 | 0·70 | 0·98 | 0·032 |
| 8 | rs11057405 | 0·82 | 0·69 | 0·97 | 0·026 | 71 | rs7899106 | 0·83 | 0·70 | 0·98 | 0·034 |
| 9 | rs11165643 | 0·83 | 0·70 | 0·98 | 0·036 | 72 | rs7903146 | 0·82 | 0·69 | 0·97 | 0·025 |
| 10 | rs1167827 | 0·82 | 0·69 | 0·97 | 0·026 | 73 | rs887912 | 0·81 | 0·69 | 0·96 | 0·017 |
| 11 | rs11727676 | 0·83 | 0·70 | 0·98 | 0·033 | 74 | rs9374842 | 0·82 | 0·69 | 0·97 | 0·021 |
| 12 | rs12286929 | 0·82 | 0·69 | 0·97 | 0·023 | 75 | rs9400239 | 0·82 | 0·69 | 0·97 | 0·021 |
| 13 | rs12401738 | 0·83 | 0·70 | 0·99 | 0·037 | 76 | rs9540493 | 0·82 | 0·69 | 0·97 | 0·021 |
| 14 | rs12429545 | 0·83 | 0·70 | 0·98 | 0·032 | 77 | rs977747 | 0·82 | 0·69 | 0·97 | 0·021 |
| 15 | rs12940622 | 0·83 | 0·70 | 0·98 | 0·033 |  |  |  |  |  |  |
| 16 | rs13021737 | 0·80 | 0·67 | 0·95 | 0·014 |  |  |  |  |  |  |
| 17 | rs13078960 | 0·83 | 0·70 | 0·99 | 0·037 |  |  |  |  |  |  |
| 18 | rs13107325 | 0·85 | 0·72 | 1·00 | 0·050 |  |  |  |  |  |  |
| 19 | rs13191362 | 0·82 | 0·69 | 0·97 | 0·021 |  |  |  |  |  |  |
| 20 | rs13201877 | 0·82 | 0·69 | 0·98 | 0·029 |  |  |  |  |  |  |
| 21 | rs1441264 | 0·83 | 0·70 | 0·98 | 0·034 |  |  |  |  |  |  |
| 22 | rs1460676 | 0·83 | 0·70 | 0·98 | 0·031 |  |  |  |  |  |  |
| 23 | rs1516725 | 0·83 | 0·70 | 0·99 | 0·038 |  |  |  |  |  |  |
| 24 | rs1528435 | 0·82 | 0·70 | 0·98 | 0·031 |  |  |  |  |  |  |
| 25 | rs1558902 | 0·84 | 0·70 | 1·00 | 0·059 |  |  |  |  |  |  |
| 26 | rs16851483 | 0·83 | 0·70 | 0·99 | 0·039 |  |  |  |  |  |  |
| 27 | rs16951275 | 0·82 | 0·69 | 0·98 | 0·031 |  |  |  |  |  |  |
| 28 | rs17001654 | 0·84 | 0·72 | 0·99 | 0·045 |  |  |  |  |  |  |
| 29 | rs17024393 | 0·82 | 0·69 | 0·98 | 0·031 |  |  |  |  |  |  |
| 30 | rs17094222 | 0·83 | 0·70 | 0·98 | 0·035 |  |  |  |  |  |  |
| 31 | rs17203016 | 0·83 | 0·70 | 0·98 | 0·031 |  |  |  |  |  |  |
| 32 | rs17405819 | 0·82 | 0·69 | 0·97 | 0·027 |  |  |  |  |  |  |
| 33 | rs17724992 | 0·83 | 0·70 | 0·98 | 0·032 |  |  |  |  |  |  |
| 34 | rs1808579 | 0·83 | 0·70 | 0·99 | 0·037 |  |  |  |  |  |  |
| 35 | rs1928295 | 0·82 | 0·69 | 0·98 | 0·030 |  |  |  |  |  |  |
| 36 | rs2033529 | 0·82 | 0·69 | 0·98 | 0·028 |  |  |  |  |  |  |
| 37 | rs2033732 | 0·83 | 0·70 | 0·98 | 0·035 |  |  |  |  |  |  |
| 38 | rs205262 | 0·82 | 0·69 | 0·97 | 0·026 |  |  |  |  |  |  |
| 39 | rs2112347 | 0·82 | 0·69 | 0·98 | 0·030 |  |  |  |  |  |  |
| 40 | rs2121279 | 0·82 | 0·69 | 0·97 | 0·021 |  |  |  |  |  |  |
| 41 | rs2176040 | 0·83 | 0·70 | 0·98 | 0·032 |  |  |  |  |  |  |
| 42 | rs2176598 | 0·82 | 0·69 | 0·97 | 0·025 |  |  |  |  |  |  |
| 43 | rs2207139 | 0·82 | 0·69 | 0·97 | 0·023 |  |  |  |  |  |  |
| 44 | rs2287019 | 0·82 | 0·69 | 0·97 | 0·025 |  |  |  |  |  |  |
| 45 | rs2365389 | 0·82 | 0·69 | 0·98 | 0·030 |  |  |  |  |  |  |
| 46 | rs2820292 | 0·83 | 0·70 | 0·99 | 0·038 |  |  |  |  |  |  |
| 47 | rs2836754 | 0·82 | 0·69 | 0·97 | 0·026 |  |  |  |  |  |  |
| 48 | rs29941 | 0·82 | 0·69 | 0·98 | 0·027 |  |  |  |  |  |  |
| 49 | rs3101336 | 0·84 | 0·70 | 0·99 | 0·045 |  |  |  |  |  |  |
| 50 | rs3736485 | 0·82 | 0·69 | 0·97 | 0·025 |  |  |  |  |  |  |
| 51 | rs3817334 | 0·82 | 0·69 | 0·98 | 0·031 |  |  |  |  |  |  |
| 52 | rs3849570 | 0·81 | 0·69 | 0·96 | 0·019 |  |  |  |  |  |  |
| 53 | rs3888190 | 0·85 | 0·72 | 1·00 | 0·055 |  |  |  |  |  |  |
| 54 | rs4256980 | 0·83 | 0·70 | 0·98 | 0·031 |  |  |  |  |  |  |
| 55 | rs4740619 | 0·83 | 0·70 | 0·99 | 0·037 |  |  |  |  |  |  |
| 56 | rs4787491 | 0·83 | 0·70 | 0·99 | 0·037 |  |  |  |  |  |  |
| 57 | rs492400 | 0·83 | 0·70 | 0·98 | 0·033 |  |  |  |  |  |  |
| 58 | rs543874 | 0·80 | 0·68 | 0·95 | 0·015 |  |  |  |  |  |  |
| 59 | rs6091540 | 0·83 | 0·70 | 0·98 | 0·035 |  |  |  |  |  |  |
| 60 | rs6465468 | 0·83 | 0·70 | 0·98 | 0·034 |  |  |  |  |  |  |
| 61 | rs6477694 | 0·82 | 0·69 | 0·98 | 0·027 |  |  |  |  |  |  |
| 62 | rs6567160 | 0·84 | 0·71 | 1·00 | 0·054 |  |  |  |  |  |  |
| 63 | rs657452 | 0·83 | 0·70 | 0·98 | 0·032 |  |  |  |  |  |  |

Legend: SNP = single nucleotide polymorphism; OR = odds ratio; LCI = lower confidence interval; UCI = upper confidence interval

S1 Fig: Funnel plot to show symmetrical distribution of individual variant estimates around the point estimate

Legend: Funnel plot of MR estimate based on individual variants plotted against the inverse of their standard error. The highest point is rs1558902 (*FTO*), which has the smallest standard error (see forest plot).

S2 Fig: Forest plot using 97 ‘un-clumped’ independent SNPs from Locke et al. Nature 2015 [17]


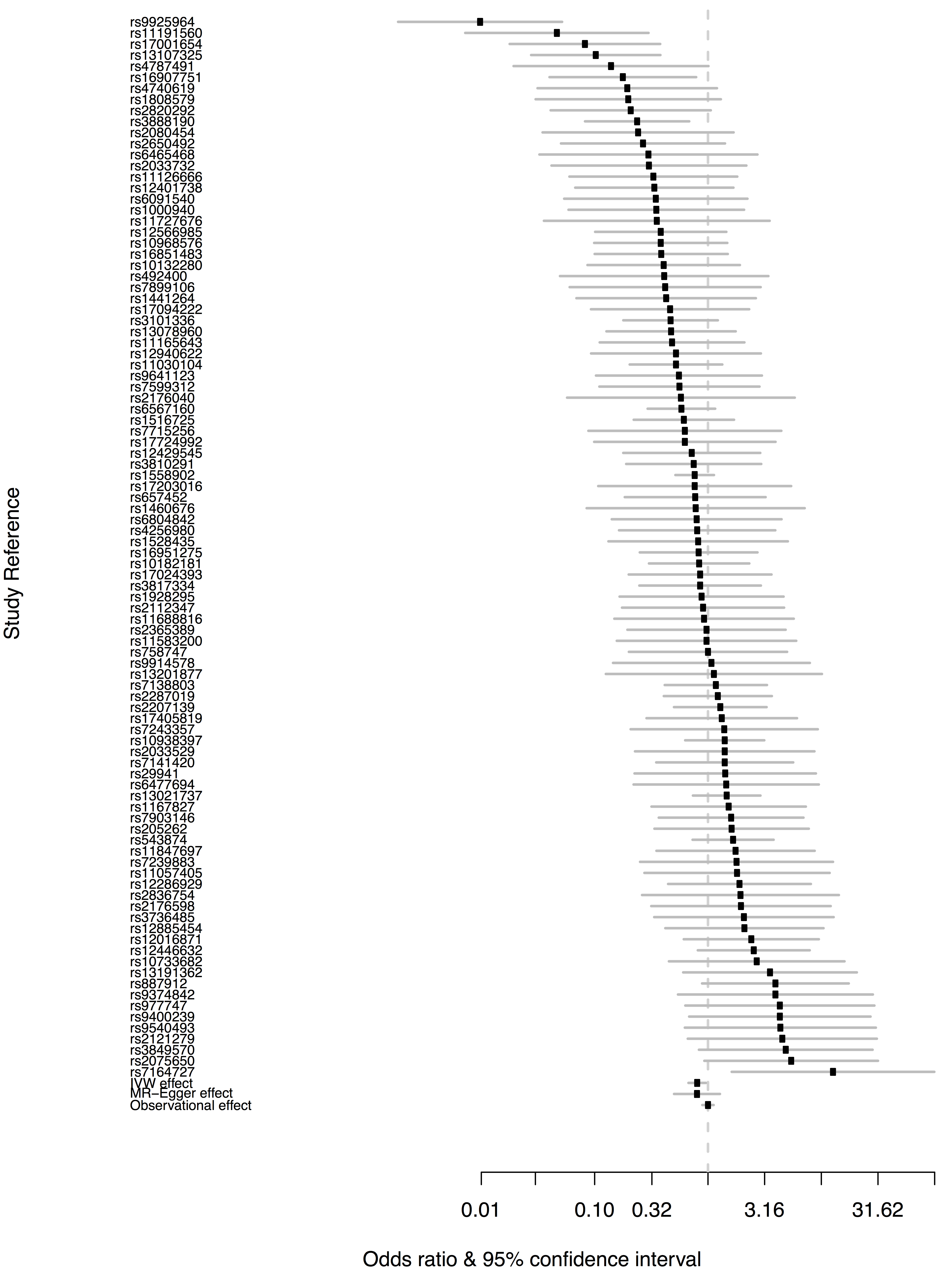


Legend: 96 variants included here because a proxy was not available for 1 of the original 97. Ratios for individual SNPs are listed according to magnitude of effect in instrumental variable analysis and are presented with pooled effects using IVW methods and MR-Egger regression. The most recent meta-analysis of observational studies is also plotted (Wang et al. 2015 [13]). Squares represent the point estimate and the bars are the 95% confidence intervals. The IVW OR was 0.80 (95% CI 0.67-0.97) and the MR-Egger OR 0.80 (95% CI 0.51-1.26), with an intercept of 0.0001 (p=0.98).

S3 Fig: Association of individual SNPs with BMI and Parkinson's disease risk


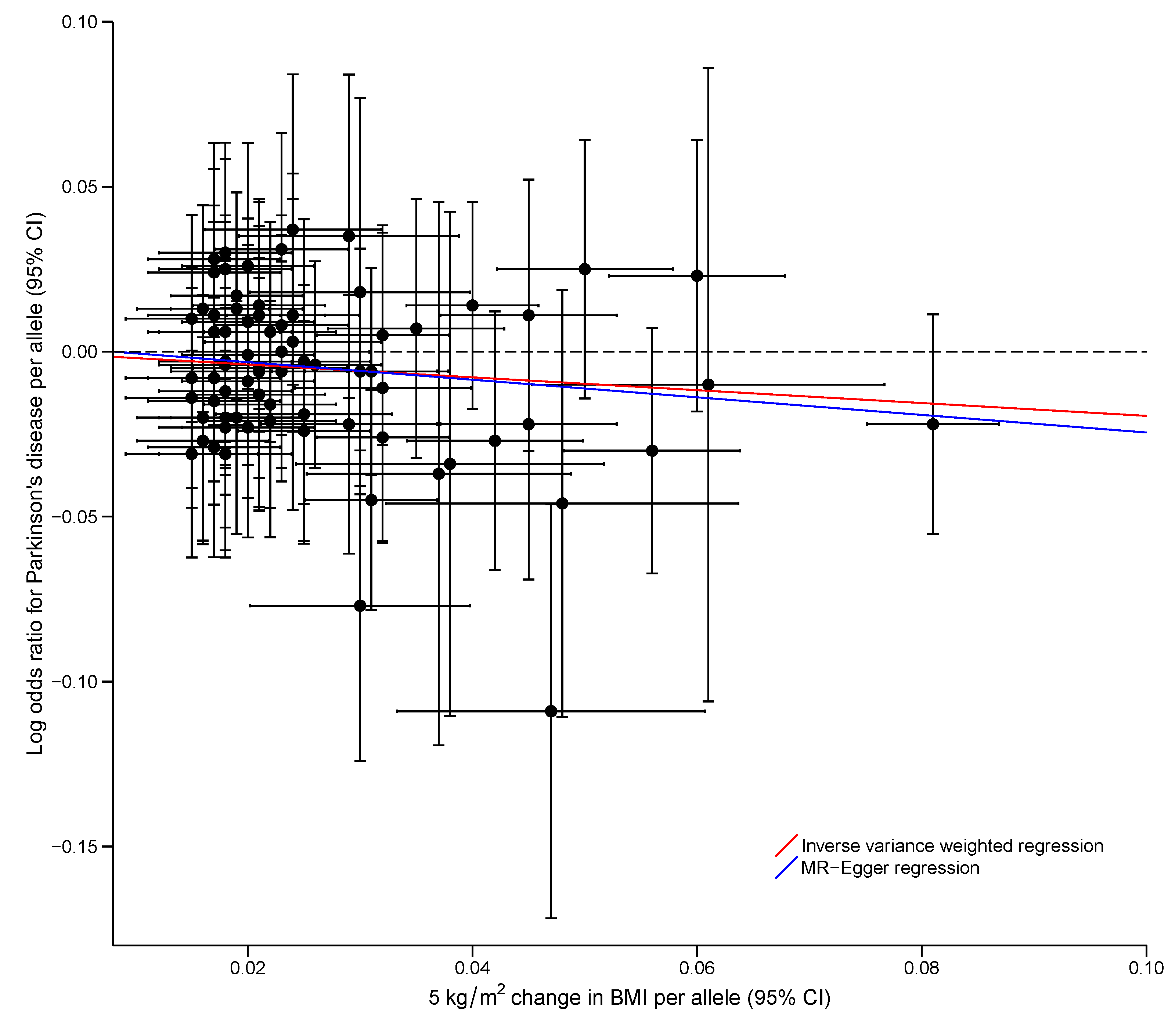
Legend: The associations are from the GIANT consortium and IPDGC meta-analyses for the 77 SNPs in the genetic instrument. Error bars represent 95% confidence intervals. The gradients of the red and blue regression lines correspond to the instrumental variable estimates of the effect of BMI on Parkinson's disease risk with the inverse variance weighted method and MR-Egger method, respectively.

Frailty analysis: Simulations to assess the contribution of survival bias to protective associations of body mass index and Parkinson's disease

## Simulation strategy

We construct a model whereby Parkinson's disease (PD) is not biologically related to body mass index (BMI), but BMI is associated with mortality, and PD is associated with age

BMI ~ snp(s)
mortality ~ age + BMI
PD ~ age

We simulated a large population ($n=500000$) where each individual has alleles at 77 BMI associated SNPs (Locke et al. 2015) (using only LD independent SNPs out of the reported 97, r-square cut-off 0.001 within 10Mb windows). PD status, age values, BMI values, and alive/dead status are also simulated.

Age values are generated to match the reported age distributions in (M. A. Nalls et al. 2014). BMI SNPs are generated as a function of their allele frequencies, such that for individual $i$ at SNP $j$ their genotype value is $g_{\mathrm{ij}} Binom(2,p_{j})$ where $p_{j}$ is the allele frequency of SNP $j$. The BMI values are a function of the BMI SNPs, such that

$$x_{i}=\sum g_{\mathrm{ij}}\beta_{j}+e_{j}$$

where $e_{j}\sim N(0,V_{E})$, where the genetic variance $V_{G}=\sum2p_{j}(1-p_{j})\beta_{j}^{2}$ and residual variance $V_{E}=V_{P}-V_{G}$. The phenotypic variance, $V_{P}$, is the variance of BMI that was used to obtain the effect sizes.

PD status was simulated as a function of age, based on age related incidence obtained from (Driver et al. 2009). Alive/dead status was modelled as a function of age and BMI values. The baseline survival function was generated from the Gompertz-Makeham law of mortality, with age related hazard function

$$h(t)=aexp(bt)+\lambda$$

which has CDF

$$F(t)=1-exp(-\lambda t-\frac{a}{b}(e^{\mathrm{bt}}-1))$$

giving the baseline survival function:

$$S_{b}(t)=1-F(t)=-exp(-\lambda t-\frac{a}{b}(e^{\mathrm{bt}}-1))$$

The influence of BMI on survival is then incorporated into the full survival model as

$$S(t)=S_{b}(t)^{w(x)}$$

where $x$ is the BMI value and $w(x)$ is a function that uses external data to relate BMI with mortality. Following (Davey Smith et al. 2009), a causal effect on all-cause mortality hazard ratios of 1.16 per standard deviation increase in BMI was simulated.

Once these simulated data are generated, only 'alive' individuals are retained, and individuals are then selected for MR analysis. 13,708 PD individuals and 95,282 non-PD individuals are sampled based on the distribution of ages of cases and controls in the original PD GWAS (Nalls et al. 2014), such that the mean age of the cases are 60.6 years old, and of controls are 53.1 years old. Finally, two-sample MR analysis is applied to the selected individuals from these simulated data.

This procedure is repeated 1000 times to obtain an empirical distribution of frailty effects for the given parameters.

We also performed an extra set of simulations to evaluate whether the frailty estimates change if BMI itself is also influenced by age. In order to model this we use BMI life course trajectories as presented by Dahl et al (2014). Supplementary figure 4 shows that if BMI is influenced by age and SNPs then 2-sample MR estimates are not changed compared to the original model of BMI being only influenced by age.

**References**

Davey Smith G, Sterne JAC, Fraser A, et al. The association between BMI and mortality using offspring BMI as an indicator of own BMI: large intergenerational mortality study. *BMJ* 2009; **339**: b5043.

Dahl AK, Reynolds CA, Fall T, Magnusson PK, Pedersen NL. Multifactorial analysis of changes in body mass index across the adult life course: a study with 65 years of follow-up*. Int J Obes (Lond)*. 2014 Aug;38(8):1133-41.

Driver JA, Logroscino G, Gaziano JM, et al. Incidence and remaining lifetime risk of Parkinson disease in advanced age. *Neurology* 2009; **72** : 432–38.

Locke AE, Kahali B, Berndt SI, et al. Genetic studies of body mass index yield new insights for obesity biology. *Nature* 2015; **518**: 197–206.

Nalls, MA, Pankratz N, Lill CM, et al. Large-scale meta-analysis of genome-wide association data identifies six new risk loci for Parkinson’s disease. *Nature Genetics* 2014; **46** : 989–993.

**S4 Fig: Comparison of frailty estimates for different models of BMI**

Legend: Comparison of two models for frailty simulations, one where BMI is modelled to be influenced only by SNPs, and another where BMI is modelled to be influenced by SNPs and age, where age has an effect to match the trajectories shown by Dahl et al 2014 (https://www.ncbi.nlm.nih.gov/pubmed/24193660). There is a clear influence of age influencing BMI on the observational association, but the MR estimates are not influenced by age influencing BMI.

**S5 Table: Cohorts included in the GIANT consortium meta-analysis (adapted from Locke et al. Nature 2015; 518: 197–206).**

|  |  |  |  | **Ages (years)** | | | | | | **BMI (kg/m^2^)** | | | | | |  |
| --- | --- | --- | --- | --- | --- | --- | --- | --- | --- | --- | --- | --- | --- | --- | --- | --- |
| **Study** | **Full name** | **Study design** | **Total sample size** | **Men** | | | **Women** | | | **Men** | | | **Women** | | | **References** |
|  |  |  | *N* | *N* | Mean | SD | *N* | Mean | SD | *N* | Mean | SD | *N* | Mean | SD |  |
| AGES | Age, Gene/Environment Susceptibility-Reykjavik Study | Population-based | 3,219 | 1,352 | 49.69 | 5.87 | 1,867 | 52.00 | 6.54 | 1,351 | 25.62 | 3.09 | 1,856 | 24.89 | 3.81 | [PMID: 17351290] |
| Amish HAPI Heart Study | Amish Heredity and Phenotype Intervention Heart Study | Founder population | 915 | 468 | 45.90 | 16.60 | 437 | 47.50 | 15.10 | 468 | 26.30 | 3.50 | 437 | 28.50 | 5.70 | [PMCID: 2443415] [PMID: 18779467] |
| ARIC | Atherosclerosis Risk in Communities Study | Population-based | 15,792 | 3,822 | 54.69 | 5.70 | 4,286 | 53.97 | 5.67 | 3,822 | 27.48 | 4.01 | 4,286 | 26.63 | 5.52 | [PMID: 2646917] [PMID: 19557197] |
| B58C (T1DGC) | British 1958 birth cohort (Type 1 Diabetes Genetic Consortium controls) | Population-based | 2,592 | 1,259 | 45.31 | 0.34 | 1,328 | 45.27 | 0.34 | 1,259 | 28.02 | 4.19 | 1,328 | 26.97 | 5.58 | [PMID: 17255346] [PMID: 19430480] |
| B58C (WTCCC) | British 1958 birth cohort (Wellcome Trust Case Control Consortium controls) | Population-based | 1,502 | 741 | 44.89 | 0.34 | 738 | 44.89 | 0.35 | 741 | 27.84 | 4.29 | 738 | 26.92 | 5.44 | [PMID: 17554300] [PMID: 16155052] |
| BRIGHT | The British Genetics of Hypertension (BRIGHT) Study | Hypertension case series | 2,000 | 719 | 56.29 | 11.15 | 1,087 | 57.43 | 11.23 | 719 | 27.74 | 3.28 | 1,087 | 27.36 | 4.04 | [PMID: 17554300] [PMID: 12826435] |
| CAD_WTCCC | WTCCC Coronary Heart Disease cases | Case series | 2,000 | 1,489 | 59.97 | 7.98 | 387 | 60.30 | 8.47 | 1,489 | 27.55 | 3.91 | 387 | 27.84 | 5.23 | [PMID: 17554300] |
| CAPS1 Cases | Cancer Prostate in Sweden 1 | Case/control study of prostate cancer | 505 | 505 | 68.15 | 7.38 | NA | NA | NA | 484 | 26.42 | 3.48 | NA | NA | NA | [PMID: 18073375] |
| CAPS1 Controls | Cancer Prostate in Sweden 1 | Case/control study of prostate cancer | 506 | 506 | 66.36 | 7.50 | NA | NA | NA | 483 | 26.49 | 3.58 | NA | NA | NA | [PMID: 18073375] |
| CAPS2 Cases | Cancer Prostate in Sweden 2 | Case/control study of prostate cancer | 1,483 | 1,483 | 66.13 | 7.07 | NA | NA | NA | 1,423 | 26.34 | 3.37 | NA | NA | NA | [PMID: 18073375] |
| CAPS2 Controls | Cancer Prostate in Sweden 2 | Case/control study of prostate cancer | 519 | 519 | 67.24 | 7.35 | NA | NA | NA | 500 | 26.03 | 3.32 | NA | NA | NA | [PMID: 18073375] |
| CHS | Cardiovascular Health Study | Population-based cohort study | 3,228 | 1,281 | 73.00 | 5.66 | 1,957 | 71.90 | 5.15 | 1,276 | 26.40 | 3.50 | 1,952 | 26.40 | 4.78 | [PMID: 1669507] |
| CoLaus | Cohorte Lausannoise | Population-based | 6,188 | 2,547 | 52.92 | 10.77 | 2,862 | 53.88 | 10.72 | 2,547 | 26.64 | 4.19 | 2,861 | 25.15 | 4.91 | [PMID 18366642] |
| CROATIA | VIS (EUROSPAN) and KORCULA | Population-based | 795 | 328 | 55.95 | 14.94 | 467 | 56.97 | 15.64 | 328 | 27.55 | 3.69 | 467 | 27.18 | 4.50 | [PMID: 18952825  [PMID: 19798445] |
| deCODE | deCODE genetics sample set | Population-based | 38,446 | 9,213 | 64.74 | 15.93 | 17,586 | 57.94 | 18.46 | 9,213 | 27.71 | 4.70 | 17,586 | 26.83 | 5.49 | [PMID: 19079260] |
| DGI (cases) | Diabetes Genetics Initiative of Broad Institute of Harvard and MIT, Lund University, and Novartis Institutes of BioMedical Research | Case/control study of diabetes | 1,658 | 688 | 63.42 | 9.91 | 629 | 65.20 | 10.12 | 688 | 28.09 | 3.83 | 629 | 28.62 | 4.80 | [PMID: 17463246] |
| DGI (controls) | Diabetes Genetics Initiative of Broad Institute of Harvard and MIT, Lund University, and Novartis Institutes of BioMedical Research | Case/control study of diabetes | 1,595 | 553 | 58.08 | 9.74 | 535 | 58.40 | 9.40 | 553 | 26.76 | 3.22 | 535 | 26.76 | 4.11 | [PMID: 17463246] |
| EGCUT | Estonian Genome Center, University of Tartu | Population-based | 1,412 | 697 | 40.62 | 16.78 | 720 | 42.88 | 15.93 | 697 | 26.05 | 4.61 | 720 | 26.25 | 6.02 | [PMID: 19424496]  [PMID: 15133739] |
| EPIC-Obesity Study | European Prospective Investigation into Cancer and Nutrition - Obesity Study | Population-based | 2,566 | 1,131 | 59.82 | 9.02 | 1,284 | 58.74 | 9.00 | 1,131 | 26.62 | 3.21 | 1,284 | 26.19 | 4.39 | [PMID: 10466767]  [PMID: 18454148] |
| Fenland | Fenland Study | Population-based | 1,500 | 615 | 44.48 | 7.32 | 787 | 45.34 | 7.18 | 615 | 27.62 | 4.07 | 787 | 26.68 | 5.46 | [PMID: 19079261] |
| FRAM | Framingham Heart Study | Population-based, multi-generational | 9,274 | 3,706 | 38.72 | 8.73 | 4,388 | 38.22 | 8.64 | 3,706 | 27.08 | 4.18 | 4,388 | 24.89 | 5.26 | [PMID 14025561]  [PMID 1208363]  [PMID 17372189] |
| FTC | Finnish Twin Cohort | Monozygotic twins | 152 pairs | NA | NA | NA | 125 | 63.78 | 11.68 | NA | NA | NA | 125 | 25.07 | 3.41 | [PMID: 19060911] |
| FUSION cases | Finland-United States Investigation of NIDDM Genetics | Case/control study of diabetes | 1,161 | 623 | 62.06 | 7.33 | 469 | 63.66 | 7.75 | 623 | 29.44 | 4.02 | 469 | 31.20 | 5.25 | [PMID: 17463248] |
| FUSION controls | Finland-United States Investigation of NIDDM Genetics | Case/control study of diabetes | 1,174 | 572 | 63.41 | 7.62 | 599 | 63.71 | 7.27 | 572 | 27.02 | 3.53 | 599 | 27.24 | 4.15 | [PMID: 17463248] |
| GENMETS cases | Health 2000 / GENMETS substudy | Case/control study of Metabolic syndrome | 932 | 425 | 49.19 | 10.42 | 432 | 52.36 | 11.71 | 425 | 29.45 | 3.61 | 432 | 29.70 | 4.94 | http://www.terveys2000.fi/indexe.html |
| GENMETS controls | Health 2000 / GENMETS substudy | Case/control study of Metabolic syndrome | 948 | 401 | 48.91 | 10.15 | 423 | 48.59 | 10.17 | 401 | 25.41 | 3.08 | 423 | 25.35 | 3.15 | http://www.terveys2000.fi/indexe.html |
| GerMiFSI | German Myocard Infarct Family Study I | Case/control study of MI (only Cases) | 875 | 394 | 57.27 | 8.57 | 206 | 60.39 | 8.67 | 394 | 27.36 | 3.30 | 206 | 27.17 | 4.17 | [PMID:17634449] |
| GerMiFSII | German Myocard Infarct Family Study II | Case/control study of MI (only Cases) | 1,222 | 901 | 60.14 | 12.17 | 223 | 62.80 | 12.76 | 901 | 27.82 | 3.54 | 223 | 28.06 | 4.76 | [PMID: 19198612] |
| KORA3 | Cooperative Health Research in the Region of Augsburg, KOoperative Gesundheitsforschung in der Region Augsburg | Population-based | 1,644 | 813 | 52.96 | 10.09 | 831 | 52.09 | 10.08 | 813 | 27.69 | 3.45 | 829 | 26.98 | 4.64 | [PMID 20031538] |
| KORA4 | Cooperative Health Research in the Region of Augsburg, KOoperative Gesundheitsforschung in der Region Augsburg | Population-based | 1,814 | 884 | 54.22 | 8.92 | 930 | 53.62 | 8.80 | 883 | 27.99 | 3.91 | 928 | 27.49 | 5.07 | [PMID 16032514] |
| MICROS | MICROS (EUROSPAN) | Population-based | 1,098 | 475 | 45.09 | 15.67 | 622 | 45.38 | 16.41 | 475 | 26.07 | 3.96 | 622 | 25.28 | 5.32 | [PMID: 17550581]  [PMID: 18952825] [PMID: 19798445] |
| Migen (cases) | Myocardial Infarction Genetics Consortium | Case/control of early onset MI | 1,420 | 800 | 44.53 | 4.77 | 474 | 49.66 | 7.28 | 775 | 28.79 | 4.99 | 470 | 28.97 | 7.42 | [PMID: 19198609] |
| Migen (controls) | Myocardial Infarction Genetics Consortium | Case/control of early onset MI | 1,558 | 846 | 46.23 | 8.41 | 561 | 49.22 | 7.50 | 844 | 27.12 | 4.08 | 558 | 27.07 | 6.65 | [PMID: 19198609] |
| NBS_WTCCC | WTCCC National Blood Service donors | Population-based | 1,500 | 694 | 45.46 | 11.75 | 743 | 41.44 | 12.59 | 694 | 26.76 | 4.12 | 743 | 25.75 | 4.46 | [PMID: 17554300] |
| NFBC-1966 | Northern Finland Birth Cohorts 1966 | Population-based | 5,654 | 2,250 | 31.00 | 0.00 | 2,247 | 31.00 | 0.00 | 2,250 | 25.18 | 3.62 | 2,247 | 24.16 | 4.68 | [PMID: 19060910] |
| NHS | The Nurses' Health Study | Nested case/control | 2,368 | NA | NA | NA | 2,265 | 54.46 | 6.45 | NA | NA | NA | 2,265 | 25.13 | 4.53 | [PMID: 17529973] |
| NSPHS | Northern Sweden Population Health Study (EUROSPAN) | Population-based | 720 | 309 | 47.56 | 20.83 | 347 | 46.47 | 20.60 | 307 | 26.75 | 4.54 | 340 | 25.97 | 5.07 | [PMID: 18952825] [PMID: 19798445] |
| NTRNESDA cases | Netherlands Twin Register & the Netherlands Study of Depression and Anxiety | Case/control study of depression and anxiety | 1,860 | 527 | 44.65 | 11.84 | 1,200 | 41.62 | 12.77 | 527 | 26.42 | 4.43 | 1,200 | 25.41 | 5.31 | [PMID: 18763692]  [PMID: 18197199] |
| NTRNESDA controls | Netherlands Twin Register & the Netherlands Study of Depression and Anxiety | Case/control study of depression and anxiety | 1,860 | 683 | 47.17 | 14.45 | 1,106 | 43.72 | 13.63 | 683 | 25.76 | 3.46 | 1,106 | 24.86 | 4.22 | [PMID: 18763692] [PMID: 18197199] |
| ORCADES | Orkney Complex Disease Study (EUROSPAN) | Population-based | 719 | 332 | 54.27 | 15.73 | 384 | 53.01 | 15.68 | 332 | 28.08 | 4.27 | 384 | 27.48 | 5.18 | [PMID: 18952825]  [PMID: 19798445] |
| PLCO | Prostate, Lung, Colorectal, and Ovarian Cancer Screening Trial | Population-based | 2,298 | 2,238 | 64.2 | 5.1 | NA | NA | NA | 2,238 | 27.5 | 3.8 | NA | NA | NA | [PMID: 17401363] |
| RS-I | Rotterdam Study I | Population-based | 7,983 | 2,427 | 68.13 | 8.16 | 3,547 | 70.32 | 9.60 | 2,372 | 25.68 | 2.99 | 3,372 | 26.74 | 4.10 | [PMID: 11753597]  [PMID:19700477]  [PMID:19728115]  [PMID:1833235] |
| RUNMC | Nijmegen Bladder Cancer Study and Nijmegen Biomedical Study | Case-study and Population-based study | 3,081 | 1,777 | 63.47 | 8.34 | 1,096 | 55.41 | 11.14 | 1,777 | 25.98 | 3.66 | 1,096 | 25.44 | 4.26 | [PMID: 17568781] [PMID: 18794855] |
| SASBAC Cases | Swedish And Singapore Breast Association Consortium | Case/control study of breast cancer | 803 | NA | NA | NA | 795 | 62.64 | 6.26 | NA | NA | NA | 793 | 25.79 | 4.00 | [PMID: 10209946] [PMID: 17132159] |
| SASBAC Controls | Swedish And Singapore Breast Association Consortium | Case/control study of breast cancer | 764 | NA | NA | NA | 764 | 62.77 | 6.34 | NA | NA | NA | 755 | 25.52 | 4.10 | [PMID: 10209946]  [PMID: 17132159] |
| SEARCH/UKOPS | Studies of Epidemiology and Risk factors in Cancer Heredity / UK Ovarian Cancer Population Study | Population-based | 1,710 | NA | NA | NA | 1,710 | 57.15 | 10.20 | NA | NA | NA | 1,556 | 26.99 | 5.20 | [PMID: 19648919] |
| SHIP | Study of Health in Pomerania | Population-based | 4,310 | 2,019 | 50.88 | 16.43 | 2,073 | 48.58 | 16.02 | 2,019 | 27.68 | 4.04 | 2,073 | 26.92 | 5.31 | [PMID: 20167617] |
| T2D_WTCCC | WTCCC Type 2 Diabetes cases | Case series | 1,999 | 1,105 | 58.95 | 9.91 | 798 | 57.94 | 10.45 | 1,105 | 30.29 | 5.36 | 798 | 32.56 | 6.87 |  |
| AE | Athero-Express Biobank Study | Case series of atherosclerotic patients | 2,512 | 469 | 68.10 | 8.90 | 217 | 68.20 | 9.60 | 433 | 26.30 | 3.20 | 189 | 26.40 | 4.70 | [PMID: 15678794] [PMID: 20720431] |
| ASCOT | Anglo-Scandinavian Cardiac Outcome Trial | Randomised control clinical trial | 3,868 | 3,122 | 63.52 | 8.17 | 673 | 64.68 | 7.64 | 3,122 | 28.93 | 4.55 | 673 | 29.47 | 5.67 | [PMID: 11685901] |
| BLSA | Baltimore Longitudinal Study on Aging | Population-based | 848 | 462 | 71.31 | 15.70 | 382 | 66.55 | 17.15 | 462 | 27.57 | 4.52 | 382 | 25.93 | 4.83 |  |
| BSN (BHS) | Busselton Health Study | Population-based | 1,366 | 558 | 53.47 | 17.15 | 769 | 53.720 | 17.076 | 558 | 26.62 | 3.57 | 769 | 25.490 | 4.420 |  |
| COROGENE | Genetic Predisposition of Coronary Heart Disease in Patients Verified with Coronary Angiogram | Population-based | 4,130 | 2,266 | 59.66 | 12.83 | 1,490 | 62.61 | 13.47 | 2,265 | 27.39 | 4.23 | 1,491 | 26.87 | 5.21 | [PMID: 19820697] |
| DESIR | Data from an Epidemiological Study on the Insulin Resistance syndrome | Population-based | 731 | 178 | 52.65 | 5.59 | 538 | 49.03 | 8.55 | 178 | 23.15 | 1.16 | 538 | 21.36 | 1.85 | [PMID: 8927780] |
| DNBC | Danish National Birth Cohort - Preterm Delivery Study | Case/control study of preterm delivery | 1,937 | NA | NA | NA | 1,802 | 29.31 | 4.20 | NA | NA | NA | 1,802 | 23.52 | 4.31 | [PMID: 11775787] [PMID: 21885063] |
| EGCUT-370 | Estonian Genome Center, University of Tartu | Population-based | 866 | 416 | 37.00 | 15.60 | 450 | 37.30 | 15.60 | 416 | 25.60 | 4.10 | 450 | 24.40 | 4.80 |  |
| EGCUT-OMNI | Estonian Genome Center, University of Tartu | Population-based | 1,356 | 517 | 46.40 | 15.80 | 839 | 67.90 | 21.60 | 517 | 26.90 | 4.40 | 839 | 27.10 | 5.40 |  |
| Erasmus Ruchphen Family Study (ERF) | Erasmus Rucphen Family Study | Family-based | 2,726 | 1,218 | 49.50 | 14.20 | 1,508 | 48.30 | 14.40 | 1,218 | 27.20 | 4.10 | 1,507 | 26.70 | 5.20 | [PMID:15054401] |
| FamHS | Family Heart Study | Population-based | 1,486 | 632 | 48.10 | 13.80 | 831 | 47.50 | 12.90 | 632 | 27.80 | 4.30 | 831 | 27.10 | 6.10 | [PMID:8651220] [PMID: 22144573] |
| FinGesture cases | Finnish Genetic Study of Arrhythmic Events | Disease cohort (MI cases only) | 1,103 | 745 | 61.19 | 10.58 | 198 | 67.44 | 10.33 | 739 | 27.22 | 3.93 | 196 | 28.14 | 5.17 |  |
| GOOD | Gothenburg Osteoporosis and Obesity Determinants Study | Population-based | 1,056 | 938 | 18.9 | 0.60 | NA | NA | NA | 938 | 22.4 | 3.20 | NA | NA | NA | [PMID: 16007330] |
| Health ABC | Health, Aging, and Body Composition Study | Longitudinal cohort study | 1,655 | 873 | 73.90 | 2.87 | 782 | 73.64 | 2.80 | 873 | 27.05 | 3.69 | 782 | 26.14 | 4.53 | PMID: 10865790 |
| HBCS | Helsinki Birth Cohort Study | Population-based birth cohort | 1,872 | 737 | 61.41 | 2.75 | 991 | 61.55 | 3.05 | 736 | 27.56 | 4.30 | 990 | 27.75 | 5.06 | [PMID: 18541567] [PMID: 15764690] |
| HERITAGE Family Study | Health, Risk Factors, Training  and Genetics (HERITAGE) Family Study | Family Study,  baseline data of an  exercise training intervention | 500 | 244 | 36.50 | 15.00 | 256 | 35.10 | 14.10 | 244 | 26.70 | 4.90 | 256 | 25.00 | 4.90 |  |
| InCHIANTI | Invecchiare in Chianti | Population-based | 1,210 | 512 | 66.60 | 15.30 | 627 | 68.30 | 15.10 | 512 | 27.00 | 3.40 | 627 | 27.30 | 4.70 | [PMID: 11129752] |
| IPM (Mount Sinai BioMe) | The Charles Bronfman Institute for Personalized Medicine BioMe Biobank Program | Hospital-based | 3,069 | 1,354 | 61 | 12 | 1,513 | 63 | 13 | 1,353 | 28.32 | 5.71 | 1,513 | 30.25 | 7.27 | [PMID:21573225] |
| LifeLines | LifeLines Cohort study | Population-based | 9,480 | 3,480 | 48.10 | 11.30 | 4,641 | 44.00 | 11.10 | 3,479 | 26.60 | 3.60 | 4,639 | 25.70 | 4.70 | [PMID:18075776] |
| LLS | Leiden Longevity Study | Family based | 2,415 | 872 | 60.00 | 6.60 | 1,049 | 58.60 | 6.60 | 869 | 25.70 | 2.91 | 1,034 | 24.90 | 3.50 | [PMID: 16251894] [PMID:19682117] |
| LOLIPOP_EW610 | London Life Sciences Prospective Population Study | Population-based | 945 | 678 | 56.10 | 9.80 | 249 | 55.70 | 9.70 | 678 | 27.80 | 4.40 | 249 | 26.60 | 5.10 | [PMID:21909110] |
| LOLIPOP_EWA | London Life Sciences Prospective Population Study | Population-based with some enrichment | 878 | 513 | 54.40 | 10.10 | NA | NA | NA | 513 | 28.30 | 4.60 | NA | NA | NA | [PMID:18940312] |
| LOLIPOP_EWP | London Life Sciences Prospective Population Study | Population-based with some enrichment | 1,006 | 651 | 55.70 | 9.10 | NA | NA | NA | 651 | 28.60 | 5.30 | NA | NA | NA | [PMID:18193046] |
| MGS | Molecular Genetics of Schizophrenia/NIMH Repository Control Sample | Population-based (survey research method) | 2,681 | 1,247 | 52.668003 | 16.01 | 1,350 | 48.479 | 16.292 | 1,247 | 30.846626 | 6.45 | 1,350 | 31.918 | 8.549 | [PMID: 19571809] [PMID: 18198266] |
| NELSON | Dutch and Belgian Lung Cancer Screening Trial | Current and former heavy smokers | 3,082 | 2,668 | 59.90 | 5.50 | NA | NA | NA | 1,135 | 27.10 | 3.60 | NA | NA | NA | [PMID: 17131307] |
| PLCO2 | Prostate, Lung, Colorectal, and Ovarian Cancer Screening Trial | Population-based case/control study of cancer | 4,219 | 2,685 | 63.90 | 5.15 | 1,477 | 63.80 | 5.25 | 2,685 | 27.25 | 4.05 | 1,477 | 26.55 | 5.05 | [PMID: 21490707] |
| PROCARDIS | Precocious Coronary Artery Disease | Case/control study of CAD | 13,000 | 5,331 | 60.40 | 8.10 | 1,947 | 62.00 | 7.60 | 5,331 | 28.00 | 4.10 | 1,947 | 28.20 | 5.50 | [PMID: 21378988]  [PMID: 18048406] |
| PROSPER/PHASE | The PROspective study of Pravastatin in the Elderly at Risk for vascular disease | Randomized controlled trial | 5,784 | 2,524 | 75.00 | 3.30 | 2,720 | 75.70 | 3.40 | 2,524 | 26.60 | 3.60 | 2,720 | 27.10 | 4.70 |  |
| QFS | Quebec Family Study | Population-based | 951 | 370 | 42.50 | 16.30 | 490 | 42.90 | 16.90 | 370 | 27.50 | 6.40 | 490 | 27.90 | 8.50 | [PMID: 3391737] |
| QIMR | Twin study at Queensland Instutite of Medical Rearch | Population-based | 11,930 | 1,470 | 44.71 | 16.34 | 2,157 | 43.97 | 15.31 | 1,470 | 26.01 | 3.93 | 2,157 | 25.03 | 5.08 | [PMID: 19896111] |
| RISC | Relationship between Insulin Sensitivity and Cardiovascular disease Study | Population-based | 1,566 | 453 | 43.30 | 8.60 | 578 | 44.50 | 8.20 | 453 | 26.40 | 3.50 | 578 | 24.80 | 4.20 | [PMID:14968294] |
| RSIII | Rotterdam Study III | Population-based | 3,932 | 877 | 55.90 | 5.40 | 1,129 | 56.20 | 6.00 | 877 | 28.00 | 4.10 | 1,129 | 27.50 | 5.10 | [PMID: 11753597] [PMID:19700477] [PMID:21877163] [PMID:1833235] |
| SHIP-TREND | Study of Health in Pomerania - TREND | Population-based | 997 | 432 | 50.13 | 14.25 | 554 | 50.13 | 13.26 | 432 | 27.81 | 3.80 | 554 | 26.99 | 5.08 | [PMID: 20167617] |
| Sorbs | Sorbs are self-contained population from Eastern Germany, European Descent | Population-based | 1,097 | 371 | 48.1 | 16.70 | 536 | 48.000 | 15.900 | 371 | 27.2 | 4.00 | 536 | 26.900 | 5.500 | [PMID: 19584900] |
| TRAILS | Tracking Adolescents' Individual Lives Survey | Population-based (measured at 18yrs of age) | 1,491 | 539 | 19.20 | 0.50 | 603 | 19.10 | 0.60 | 539 | 22.60 | 3.80 | 602 | 23.00 | 3.70 | [PMID: 18263649] |
| TWINGENE | TWINGENE | Population-based | 9,836 | 4,349 | 65.68 | 8.02 | 4,827 | 64.85 | 8.22 | 4,349 | 26.33 | 3.54 | 4,827 | 25.80 | 4.31 | [PMID: 8981957] |
| TwinsUK | TwinsUK | Twins cohort | 5,654 | NA | NA | NA | 3,003 | 47.23 | 12.81 | NA | NA | NA | 3,003 | 25.20 | 4.71 | [PMID: 22589082] |
| WGHS | Women's Genome Health Study | Population-based | 23,294 | NA | NA | NA | 22,888 | 54.69 | 7.11 | NA | NA | NA | 22,888 | 25.91 | 4.96 | [PMID: 18070814] |
| YFS | The Cardiovascular Risk in Young Finns Study | Population-based cohort | 2,443 | 1,123 | 37.55 | 5.06 | 1,320 | 37.57 | 5.01 | 908 | 26.76 | 4.29 | 1,081 | 25.32 | 5.03 |  |
| ADVANCE-CAD | Atherosclerotic Disease, VAscular FuNction, & GenetiC Epidemiology study | Population-based case/control study of CAD | 1,679 | 1,095 | 63.65 | 5.55 | 463 | 65.95 | 4.60 | 1,095 | 28.75 | 4.65 | 463 | 28.50 | 6.30 | [PMID:16490908] [PMID 16840522]  [PMID 17084253]  [PMID 18443000] |
| AMC-PAS | Academic Medical Centre Amsterdam Premature Atherosclerosis Study | CAD cases | 706 | 368 | 43.50 | 5.10 | 122 | 42.80 | 5.60 | 368 | 27.10 | 3.80 | 122 | 26.20 | 5.10 | [PMID:19164808] |
| ARIC Metabochip | Atherosclerosis Risk in Communities | Population-based | 3,330 | 1,224 | 53.80 | 6.00 | 2,073 | 53.40 | 5.70 | 1,224 | 27.90 | 4.90 | 2,073 | 31.00 | 6.50 | [PMID: 2646917] |
| B1958C | 1958 British Birth Cohort | Population-based | 2,359 | 1,234 | 45.00 | 0.00 | 902 | 45.00 | 0.00 | 1,232 | 27.80 | 4.20 | 902 | 26.60 | 5.30 |  |
| BHS | Busselton Health Study | Case/control study of CAD | 270 | 169 | 69.70 | 8.60 | 93 | 70.50 | 9.70 | 169 | 26.70 | 3.60 | 93 | 26.30 | 4.70 | [PMID: 15486340] |
| CARDIOGENICS | CARDIOGENICS | Case/control study of CAD | 806 | 476 | 55.70 | 8.30 | 278 | 54.20 | 7.60 | 476 | 27.70 | 4.10 | 278 | 26.00 | 4.40 | [PMID: 17634449] |
| CLHNS | Cebu Longitudinal Health and Nutrition Survey (Offspring) | Population-based | 1,858 | 919 | 21.50 | 0.30 | 828 | 21.50 | 0.30 | 916 | 21.10 | 3.10 | 763 | 20.40 | 3.20 | [PMID: 20507864] [PMID: 22010046] |
| D2D 2007 | FIN-D2D study, 2007 cohort | Population-based | 2,720 | 1,284 | 61.35 | 8.10 | 1,404 | 61.25 | 7.65 | 1,284 | 28.35 | 4.35 | 1,404 | 29.20 | 5.50 | [PMID: 20459722] |
| DESIR | Data from an Epidemiological Study on the Insulin Resistance syndrome | Population-based | 4,993 | 2,108 | 46.10 | 10.20 | 1,817 | 46.20 | 10.30 | 2,081 | 25.60 | 3.40 | 1,777 | 24.80 | 4.30 | [PMID: 8927780] |
| DIAGEN | The DIAbetes GENetics Study | Clinical prevention study | 1,535 | 660 | 63.30 | 12.55 | 802 | 64.75 | 13.95 | 660 | 28.10 | 4.00 | 802 | 29.00 | 6.05 | [PMID:16801592] |
| DILGOM | Dietary, life style, and genetic determinants of obesity and metabolic syndrome | Population-based | 3,997 | 1,797 | 53.30 | 13.50 | 2,139 | 51.70 | 13.60 | 1,791 | 27.20 | 4.20 | 2,139 | 26.90 | 5.40 | [PMID: 20138944] [PMID: 20844574] |
| DPS | Diabetes Prevention Study | Population-based | 522 | 148 | 56.20 | 6.90 | 324 | 54.95 | 6.75 | 148 | 30.05 | 3.30 | 324 | 32.30 | 4.60 | [PMID: 11333990] |
| DR'S EXTRA | DR'S EXTRA | Population-based | 1,408 | 602 | 67.75 | 5.65 | 693 | 67.35 | 5.45 | 602 | 28.25 | 4.15 | 693 | 29.80 | 5.35 | [PMID: 21186108] |
| DUNDEE | DUNDEE | Population-based case/control study of diabetes | 7,544 | 3,847 | 61.70 | 10.30 | 3,140 | 61.00 | 10.60 | 3,842 | 29.20 | 4.80 | 3,134 | 29.85 | 6.05 |  |
| EAS | Edinburgh Artery Study | Population based | 733 | 353 | 64.70 | 5.60 | 378 | 64.20 | 5.70 | 353 | 25.30 | 3.10 | 378 | 25.30 | 4.20 | [PMID: 1917239] |
| EGCUT | Estonian Genome Center of University of Tartu | CAD cases, T2D cases, and controls | 2,619 | 1,063 | 60.21 | 10.88 | 1,556 | 60.75 | 9.93 | 1,061 | 28.30 | 4.07 | 1,555 | 28.84 | 4.78 | [PMID: 19424496] |
| Ely Study | MRC Ely Study | Population-based | 1,625 | 744 | 61.49 | 9.13 | 855 | 60.82 | 9.26 | 744 | 27.36 | 3.99 | 855 | 27.29 | 5.37 | [PMID: 17257284] |
| EMIL (SWABIA) | Echinococcus Multilocularis and Internal Diseases in Leutkirch (EMIL) study | Population based | NA | 808 | 45.70 | 11.40 | 908 | 46.20 | 11.20 | 808 | 27.70 | 4.70 | 908 | 26.70 | 6.30 | [PMID: 16112969] |
| EPIC-Norfolk T2D | EPIC (European Prospective Investigation into Cancer) Norfolk T2D case-Cohort Study | Population-based | 978 - cohort; 736 -T2D | 842 | 60.93 | 8.63 | 842 | 60.72 | 8.94 | 842 | 27.73 | 3.52 | 842 | 28.01 | 4.54 | [PMID: 10466767] [PMID: 18454148] |
| FBPP | Family Blood Pressure Program: GenNet and HyperGEN studies | Ascertained based on a hypertensive proband | NA | 642 | 43.09 | 12.24 | 1,139 | 44.33 | 12.67 | 641 | 28.95 | 6.65 | 1,138 | 33.39 | 8.42 | [PMID: 11799070] |
| Fenland | The Fenland Study | Population-based | 3,217 | 1,486 | 46.87 | 7.19 | 1,698 | 46.90 | 7.04 | 1,486 | 27.04 | 4.09 | 1,698 | 26.48 | 5.48 | [PMID: 20519560] |
| FUSION stage 2 | FUSION stage 2 | Case/control study of diabetes | 2,951 | 1,628 | 57.05 | 8.50 | 1,301 | 58.90 | 8.15 | 1,628 | 28.35 | 4.25 | 1,301 | 29.15 | 5.20 | [PMID: 17463248] |
| GLACIER | Gene x Lifestyle interactions And Complex traits Involved in Elevated disease Risk | Prospective cohort study | 6,311 | 2,381 | 50.10 | 8.30 | 3,666 | 49.30 | 8.80 | 2,381 | 26.08 | 3.40 | 3,666 | 25.54 | 4.39 | [PMID: 19164386] [PMID: 14660243] |
| GXE | Kingston Gene-by-environment; subset of International Collaborative Study of Hypertension in Blacks (ICSHIB) | Case-cohort (upper and lower third of BMI from a cohort) | 1,039 | 140 | 38.70 | 8.90 | 473 | 40.00 | 8.10 | 140 | 27.70 | 6.90 | 473 | 32.30 | 7.50 | [PMID:9103091] [PMID:20400458] |
| HNR | Heinz Nixdorf Recall | Population-based | 4,570 | 2,250 | 59.70 | 7.80 | 2,242 | 59.62 | 7.80 | 2,250 | 28.20 | 4.00 | 2,242 | 27.65 | 5.20 | [PMID: 16121757] [PMID: 20616309] |
| HUNT 2 | The Nord-Trøndelag Health Study 2 | Population-based, although T2D case-control samples selected for metabochip typing | 1,567 | 693 | 65.60 | 12.90 | 641 | 69.15 | 12.55 | 693 | 27.30 | 3.50 | 641 | 28.95 | 4.85 |  |
| IMPROVE | Carotid Intima Media Thickness [IMT] and IMT-Progression as Predictors of Vascular Events in a High Risk European Population | Population-based | 3,450 | 1,665 | 64.00 | 5.40 | 1,783 | 64.40 | 5.50 | 1,665 | 27.40 | 3.60 | 1,783 | 27.10 | 4.80 | [PMID: 19952003] |
| KORA S3 | Cooperative Health Research in the Region of Augsburg (third survey), KOoperative Gesundheitsforschung in der Region Augsburg (dritte Studie) | Population-based | 3,113 | 604 | 40.90 | 13.30 | 674 | 40.90 | 12.70 | 599 | 26.50 | 3.40 | 659 | 25.20 | 4.70 |  |
| KORA S4 | Cooperative Health Research in the Region of Augsburg (forth survey), KOoperative Gesundheitsforschung in der Region Augsburg (vierte Studie) | Population-based | 3,028 | 585 | 42.70 | 15.80 | 637 | 41.20 | 14.80 | 582 | 26.60 | 3.60 | 624 | 25.90 | 5.10 |  |
| Leipzig Adults | Leipzig adults | Population-based | 1,005 | 399 | 42.94 | 17.30 | 591 | 43.53 | 15.89 | 400 | 35.54 | 12.39 | 592 | 37.91 | 12.41 | [PMID: 20935630] |
| LURIC | Ludwigshafen Risk and Cardiovascular Health Study | Case/control study of CAD | 3,316 | 2,008 | 59.00 | 11.15 | 888 | 64.15 | 10.30 | 2,008 | 27.35 | 3.75 | 888 | 27.25 | 4.75 | [PMID: 11258203] |
| MEC Metabochip | Multiethnic Cohort Study | Population-based | 455 | 84 | 72.50 | 7.06 | 30 | 67.80 | 7.91 | 84 | 27.60 | 4.38 | 30 | 30.80 | 7.34 | [PMID: 10695593] |
| METSIM | Metabolic Syndrome In Men | Population-based, although T2D case-control samples selected for metabochip typing | 2,176 | 2,014 | 57.10 | 5.80 | NA | NA | NA | 2,014 | 28.30 | 4.30 | NA | NA | NA | [PMID: 19223598] |
| MORGAM | MOnica Risk, Genetics, Archiving and Monograph | CVD cases and controls from population-based follow-up cohorts. | ~4,800 | 3,910 | 59.10 | 7.90 | 615 | 57.60 | 8.75 | 3,802 | 27.20 | 3.95 | 565 | 28.35 | 5.35 |  |
| MRC NSHD | MRC National Survey of Health & Development | Birth cohort | 5,362 | 464 | 53.00 | 0.00 | 515 | 53.00 | 0.00 | 464 | 27.61 | 4.16 | 515 | 27.72 | 5.54 | [PMID: 16204333] [PMID: 23977022] |
| PIVUS | Prospective Investigation of the Vasculature in Uppsala Seniors | Population-based | 999 | 490 | 70.13 | 0.17 | 488 | 70.26 | 0.15 | 490 | 27.05 | 3.72 | 488 | 27.12 | 4.92 | [PMID: 16141402] |
| PROMIS | Pakistan Risk of Myocardial Infarction Study | Case/control study of CAD | 3,712 | 2,964 | 52.10 | 10.10 | 658 | 54.00 | 9.00 | 2,285 | 25.90 | 4.20 | 513 | 26.70 | 4.80 | [PMID: 19404752] |
| SardiNIA | SardiNIA Study on Aging | Population-based | 5,190 | 2,186 | 45.80 | 16.70 | 3,004 | 45.30 | 16.50 | 2,183 | 26.50 | 3.90 | 2,999 | 25.00 | 4.90 | [PMID: 16934002] |
| SCARFSHEEP | Stockholm Coronary Artery Risk Factors/Stockholm Heart Epidemiology Progrgramme | Case/control study of CAD | 3,077 | 2,197 | 57.00 | 7.10 | 857 | 60.20 | 7.30 | 2,197 | 26.20 | 3.50 | 857 | 25.90 | 4.40 |  |
| SPT | Spanish Town | Population-based | 1,831 | 351 | 48.00 | 14.80 | 553 | 45.60 | 13.10 | 351 | 24.30 | 4.30 | 553 | 28.40 | 6.10 | [PMID:9103091]  [PMID:20400458] |
| STR | Swedish Twin Registry | Population-based | 2,702 | 838 | 74.28 | 9.97 | 1,378 | 76.07 | 9.73 | 838 | 25.13 | 3.36 | 1,378 | 25.03 | 4.24 | [PMID: 17254424] |
| TANDEM | Seychelles Family Study (TANDEM) | Ascertained based on a hypertensive proband | NA | 205 | 46.99 | 13.46 | 287 | 49.91 | 14.39 | 201 | 26.30 | 4.53 | 281 | 29.08 | 5.79 | [PMID: 15699448] |
| THISEAS | The Hellenic study of Interactions between Snps and Eating in Atherosclerosis Susceptibility | Case/control study of CAD | 1,887 | 867 | 58.20 | 12.70 | 628 | 61.30 | 13.80 | 806 | 28.40 | 4.10 | 604 | 28.60 | 5.20 | [PMID: 20167083] |
| Tromsø | Tromsø 4 | Population-based, although T2D case-control samples selected for metabochip typing | 1,421 | 706 | 57.80 | 11.15 | 712 | 62.05 | 13.40 | 706 | 27.20 | 3.65 | 712 | 27.90 | 4.85 | [PMID: 21422063] |
| ULSAM | Uppsala Longitudinal Study of Adult Men | Population-based | 1,221 | 1,112 | 71.00 | 0.63 | NA | NA | NA | 1,112 | 26.26 | 3.40 | NA | NA | NA | [PMID: 16030278] |
| WHI Metabochip | Womens Health Initiative | Clinical trial and Observational Study | 2,078 | NA | NA | NA | 2,046 | 61.00 | 6.90 | NA | NA | NA | 2,046 | 31.40 | 6.80 | [PMID: 14575938] |
| Whitehall | The Whitehall II study | Cohort of London-based civil servants | 3,413 | 1,699 | 60.51 | 5.82 | 535 | 60.60 | 5.79 | 1,699 | 26.65 | 3.63 | 535 | 27.14 | 5.31 | [PMID: 15576467] [PMID: 21441441] |
| WTCCC-T2D | Wellcome Trust Case Control Consortium - T2D non GWAS | Case series | 1,335 | 626 | 56.30 | 10.20 | 448 | 56.40 | 11.10 | 624 | 31.20 | 5.60 | 445 | 33.80 | 7.60 |  |
| HRS | Health and Retirement Study | Population-based | 12,509 | 3,424 | 68.94 | 9.75 | 4,740 | 68.27 | 10.78 | 3,424 | 29.15 | 4.98 | 4,740 | 28.70 | 6.30 |  |

**S6 Table: Cohorts included in the IPDGC meta-analysis (adapted from Nalls et al. Nat Genet 2014; 46 : 989–993).**

| **Study (Country)** | **Cases (N)** | **Controls (N)** | **Total sample size (N)** | **Cases % female** | **Controls % female** | **Cases, age at onset, mean (SD)** | **Controls, age at ascertainment, mean (SD)** | **Case ascertainment criteria** | **Control ascertainment criteria** |
| --- | --- | --- | --- | --- | --- | --- | --- | --- | --- |
| IPDGC (Iceland, deCODE) | 604 | 4916 | 5520 | 47.8 | 56.9 | 62.2 (12.3) | 80.2 (7.5) | Clinic visit; standard UK Brain Bank criteria with a modification to allow the inclusion of cases that had a family history of PD | Absence of self report, ICD-9, and medications |
| IPDGC (France) | 985 | 1984 | 2969 | 41.2 | 33 | 48.9 (12.8) | 73.7 (5.4) | Clinic visit; standard UK Brain Bank criteria with a modification to allow the inclusion of cases that had a family history of PD | Clinic visit and self-report |
| IPDGC (Germany) | 667 | 937 | 1604 | 39.8 | 48 | 55.7 (11.5) | 47.4 (12.4) | Clinic visit; standard UK Brain Bank criteria with a modification to allow the inclusion of cases that had a family history of PD | Population controls |
| IPDGC (Netherlands) | 744 | 2019 | 2763 | 36 | 63.9 | 55.6 (11.8) | 55.7 (5.8) | Clinic visit; standard UK Brain Bank criteria with a modification to allow the inclusion of cases that had a family history of PD | Population controls |
| IPDGC (USA) | 937 | 1896 | 2833 | 40.5 | 52.8 | 57.8 (13.2) | 63.3 (10.1) | Clinic visit; standard UK Brain Bank criteria with a modification to allow the inclusion of cases that had a family history of PD | Clinic visit and self-report |
| IPDGC (UK) | 1705 | 5200 | 6905 | 43.3 | 49.5 | 64.2 (12.4) | 53 (0) | Clinic visit; standard UK Brain Bank criteria with a modification to allow the inclusion of cases that had a family history of PD | Population controls |
| 23andMe.v2 (USA, Europe) | 3261 | 29499 | 32760 | 39.5 | 41.6 | 64.2 (11.2) | 49.1 (14.9) | Self-report of PD diagnosis by clinician | Self-report of neurological disease, memory loss, tremor or family history of PD removed |
| 23andMe.v3 (USA, Europe) | 866 | 32538 | 33424 | 39 | 39.6 | 63.9 (10.9) | 43.5 (15.9) | Self-report of PD diagnosis by clinician | Self-report of neurological disease, memory loss, tremor or family history of PD removed |
| Ashkenazi Jewish (USA) | 268 | 178 | 446 | 33.2 | 57.3 | 59.9 (12.1) | 69.8 (8.8) | Clinic visit; standard UK Brain Bank criteria with a modification to allow the inclusion of cases that had a family history of PD | Medical records and self-report |
| HIHG (USA) | 574 | 619 | 1193 | 36.9 | 65.4 | 57.2 (12.03) | 69.3 (9.9) | Clinic visit; standard UK Brain Bank criteria with a modification to allow the inclusion of cases that had a family history of PD | Clinic visit and self-report |
| NGRC (USA) | 1956 | 1982 | 3938 | 33.2 | 61.3 | 58.6 (11.7) | 70.3 (14.1) | Clinic visit; standard UK Brain Bank criteria with a modification to allow the inclusion of cases that had a family history of PD | Clinic visit and self-report |
| PROGENI-GenePD (USA) | 828 | 852 | 1680 | 40.1 | 60.2 | 62.1 (10.7) | 54.9 (13.1) | Clinic visit; standard UK Brain Bank criteria with a modification to allow the inclusion of cases that had a family history of PD | Clinic visit and self-report |
| CHARGE-CHS (USA) | 107 | 3164 | 3271 | 44.9 | 61.4 | 73 (5.1) | 72.3 (5.4) | Self-report, ICD-9, and medications | Absence of self report, ICD-9, and medications |
| CHARGE-FHS (USA) | 60 | 3889 | 3949 | 41.7 | 54.6 | 76.2 (10.8) | 64.2 (12.2) | Clinic visit; standard UK Brain Bank criteria with a modification to allow the inclusion of cases that had a family history of PD | On-going surveillance and periodic physician examination |
| CHARGE-AGES-RS (Iceland) | 146 | 5609 | 5755 | 54.8 | 59 | 75 (8.2) | 69 (8.9) | Clinic visit; medication use; ICD codes | Absence of self report, ICD-9, and medications |
